# Non-zoonotic tick-borne pathogens in Western Balkan

**DOI:** 10.1186/s13071-025-06740-z

**Published:** 2025-03-14

**Authors:** Naida Kapo, Ivana Zuber Bogdanović, Ema Gagović, Daria Jurković Žilić, Ratko Sukara, Bojan Adžić, Përparim Kadriaj, Šimun Naletilić, Ani Vodica, Aleksandar Cvetkovikj, Igor Djadjovski, Aleksandar Potkonjak, Sara Savić, Snežana Tomanović, Jasmin Omeragić, Adnan Hodžić, Relja Beck

**Affiliations:** 1 Department of Veterinary Clinical Sciences, Faculty of Veterinary Medicine, University of Sarajevo, Sarajevo, Bosnia and Herzegovina; 2 Diagnostic Veterinary Laboratory, Podgorica, Montenegro; 3 Department for Bacteriology and Parasitology, Laboratory for Parasitology, Croatian Veterinary Institute, Zagreb, Croatia; 4 Institute for Medical Research, National Institute of Republic of Serbia, University of Belgrade, Belgrade, Serbia; 5 Vector Control Unit, Department of Epidemiology and Control of Infectious Diseases, Institute of Public Health, Tirana, Albania; 6 Department of Pathology, Laboratory for Pathology, Croatian Veterinary Institute, Zagreb, Croatia; 7 Animal Health Department, Food Safety and Veterinary Institute, Tirana, Albania; 8 Faculty of Veterinary Medicine Skopje, National Veterinary and Food Institute, Ss. Cyril and Methodius University in Skopje, Skopje, North Macedonia; 9 Department of Veterinary Medicine, Faculty of Agriculture, University of Novi Sad, Novi Sad, Serbia; 10 Scientific Veterinary Institute “Novi Sad”, Novi Sad, Serbia; 11 Centre for Microbiology and Environmental Systems Science (CMESS), Department of Microbiology and Ecosystem Science, University of Vienna, Vienna, Austria

**Keywords:** Hard ticks, Tick-borne pathogens, Non-zoonotic diseases, Animals, Western Balkans

## Abstract

Ixodid ticks are present throughout the Western Balkan countries, including Albania, Bosnia and Herzegovina, Croatia, Montenegro, North Macedonia and Serbia, with many species serving as vectors for pathogens of both veterinary and medical importance. We have conducted a thorough examination of existing literature, encompassing historical documents, to collect information on all documented non-zoonotic tick-borne pathogens found in ticks, pets, farm animals and wild animals across the Western Balkan region. A comprehensive review was necessary due to the scarcity and scattered nature of available data from this area. The tick fauna in the Western Balkans consists of 32 species across five genera: *Ixodes*, *Haemaphysalis*, *Dermacentor*, *Rhipicephalus* and *Hyalomma*. Various pathogens responsible for diseases in animals, including bacteria and parasites, have also been documented, many of which can cause important diseases and significant reductions in animal productivity. Initial efforts were directed towards pathogen surveillance and the characterisation of non-zoonotic tick-borne pathogens, resulting in the identification of *Theileria orientalis*, *Anaplasma bovis* and *Anaplasma marginale* in cattle, although significant gaps remain in the current research. Studies on small ruminants have been sparse, with confirmed cases of *Anaplasma ovis* and *Babesia ovis* in sheep, but no comprehensive and systematic research on pathogens in goats. In contrast, research on canine piroplasms has identified several species, including *Babesia canis* and *Babesia vulpes*. Studies on wild animals, however, have predominantly focused on wild canines and carnivores, with limited attention given to non-zoonotic pathogens. Notably, only one study has reported non-zoonotic tick-borne pathogens in artiodactyl species and wild felids. This review is a much needed overview of existing research on non-zoonotic tick-borne diseases in the Western Balkans, including the historical context, current data and research gaps. Given the significant impact of these diseases on animal health and productivity, as well as their potential biodiversity, further comprehensive studies and the establishment of national surveillance systems for tick-borne diseases are essential for a better understanding and mitigation of their impact.

**Graphical abstract:**

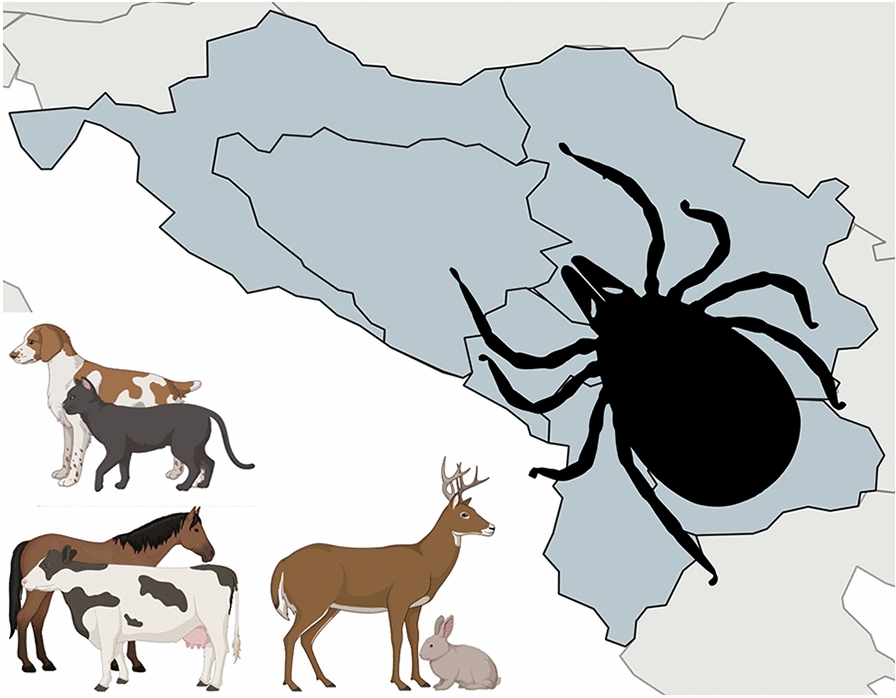

## Background

 Ticks are recognised as the primary vectors of infectious diseases in animals worldwide [1]. Over the past decades, the number of reported cases of tick-borne diseases (TBDs) in humans and animals has significantly increased [2]. Diseases that were once confined to tropical regions are now spreading to previously unaffected areas. This expansion is attributed to changes in the epidemiology of TBDs, which are linked to various factors such as climate change, increased mobility of humans and their pets, intensive animal production and greater interaction with wild animals, including habitat encroachment and recreational activities [3, 4]. In addition, the effects of climate change, global warming in particular, may significantly facilitate the expansion of ticks and extend their activity into warmer seasons. This, in turn, may contribute to an increase in the annual occurrence of TBDs [5]. Global warming specifically is a significant factor facilitating the expansion of ticks and the extension of their activity into warmer seasons.

A consideration of the impact of ticks on livestock reveals that the economic consequences of their associated TBDs, such as babesiosis, ehrlichiosis, anaplasmosis and theileriosis, are far-reaching. These diseases often present through a range of clinical signs, including fever and changes in overall health, which can lead to haemolytic anemia, anorexia and abortion—fatal conditions, particularly in young and vulnerable animals. Despite these significant adverse effects, persistent infections or mixed infections with multiple pathogens in seemingly healthy or chronically ill animals may be underestimated. This is particularly relevant as apparently healthy animals can transmit pathogens to uninfected susceptible individuals through tick bites [6].

In this context, the contemporary livestock sector in industrially advanced countries is confronted with the profound impact of various tick species and their associated diseases, which is recognised as a pivotal factor contributing to substantial productivity losses [6]. The objectives of the European Union are a targeted 50% reduction in the use of antimicrobial agents in animal and aquaculture farming by 2030, coupled with the ambitious goal of having the organic farmland encompass 25% of the total agricultural land by the same year. Thus, strategically managing Vector-borne diseases (VBDs) becomes essential for achieving the European Union's targets for organic farming and reducing antimicrobial use, ensuring long-term sustainability and economic viability in the agricultural sector. There is compelling evidence that wild animals significantly contribute to the maintenance of tick-borne pathogens (TBPs), acting as natural reservoirs for many infectious agents [7, 8]. The relevance of these natural reservoirs in the epidemiology of TBDs is currently escalating due to increases in the density of these species in some European regions over the past decades, notably red deer (*Cervus elaphus*), roe deer (*Capreolus capreolus*), wild boars (*Sus scrofa*), foxes (*Vulpes vulpes*) and wolves (*Canis lupus*) [9–11]. This trend has also fostered frequent interactions and resource-sharing among wild animals, livestock, companion animals and humans in specific areas, thereby amplifying the risk of interspecies transmission of TBPs [12].

In the Western Balkans (WBs), there is a limited understanding of the tick fauna and the pathogens they transmit, as well as of their pathogenic impact and economic significance. Similarly, current understanding of the role of wild animals in the transmission of TBDs among companion animals and livestock is rather limited. This review article aims to provide comprehensive information, including historical records, on the occurrence and distribution of non-zoonotic TBPs in ticks, companion animals, livestock and wild animals recorded in each country of the WBs. Additionally, we carefully explore and analyse the current and past situations in the field, emphasising upcoming research priorities to stimulate studies on this crucial topic and raise awareness among parasitologists, veterinarians and physicians.

## Historical overview of TBPs in animals in the Western Balkans

To obtain a comprehensive understanding of TBPs in animals in the WBs, it is essential to collect and assess historical data. In this section of the review, we provide data up to the end of the Second World War, as the redrawing of borders at that time had a significant and immediate effect on the understanding of TBDs. Through a thorough examination of historical non-English literature, we have uncovered valuable information that should be shared with the wider scientific community to enhance current understanding of TBDs in the WB region.

The earliest records on TBPs trace back to the late nineteenth century, when piroplasmosis was referred to as the 'red urination' disease, although the etiology of the causative agent was not identified at that time. This disease was prevalent across the regions of Croatia, Slovenia and Serbia in equines, bovines, caprines and ovines. Similarly, a report from the government of Bosnia and Herzegovina (1899) covering the period from 1879 to 1898 indicates that bovine piroplasmosis was consistently present in Bosnia [13]. The initial documentation on piroplasmosis in the area can be traced back to 1912, when investigations of babesiosis in sheep were being carried out in Dalmatia, Croatia. In 1921, Inchiostri [14] proposed and described the species '*Piroplasma ovis*'. This was the same time period that Bosnia and Herzegovina and Croatia were incorporated within the Austro-Hungarian Empire. Following the end of the First World War, the new entity known as the Kingdom of Slovenians, Croats and Serbs emerged, altering national boundaries and facilitating the movement of individuals and goods, including livestock. This transformation led to a significant increase in studies and reports on piroplasmosis. Petrović [15] published one of the earliest papers on piroplasmosis in 1922 as part of the work of the Antimalarial Commission, and in 1923 Đunkovski [16] described the prevalence of piroplasmosis in large animals in the area from Skopje to Ohrid, including sheep, reporting that it appeared to be a novel type of theileriosis.

According to Čolak [17], piroplasmosis was a limiting factor in the colonisation of livestock in Southern Serbia between 1920 and 1924, resulting in the death of animals, particularly those introduced from northern areas. For example, 230 sheep died in one flock near Skopje in the summer of 1923, while at least 600 cattle died in Kumanovo county in 1921. According to this author, piroplasmosis did not occur everywhere, but rather in open places where ticks are plentiful, and he questioned which of the three causative agents, *Babesiosis bovum*, Texas fever or theileriosis, were responsible for mortality, adding that “*we are still in the dark regarding aetiology*”. The local populace abandoned the importation of 'noble' cattle after realising that settlers' cattle from northern areas were more afflicted in comparison to indigenous cattle, and in a few cases, all cows died.

Because of the extremely difficult situation, the Ministry of Agriculture dispatched experts to that area to construct Veterinary Units for Disease Control in the counties of Southern Serbia [18]. Dr. Andrija Štampar, organiser of humane prophylactic medicine, encouraged the establishment of veterinary laboratories within Hygiene Departments. He recognised the importance of collaboration between veterinary and hygiene services in controlling zoonotic diseases, ensuring food safety and maintaining animal health. The attempts to reduce the harm caused by cattle diseases to farmers would have a substantial impact on the overall economic condition and health of animals in the region. The studies clearly indicated the importance of piroplasmosis as a limiting factor in the economic development of the Vardar Banovina (now North Macedonia and Southern Serbia), and “*what malaria is for humans, piroplasmosis is for animal husbandry, because imported animals died, and families would be left without income*” [19]. Piroplasmosis was described as a disease that hindered the region's development since advanced imported breeds were unable to survive, resulting in a reduction in land animal husbandry.

In 1937, Mlinac and Štrek [20] reported *Theileria parva* in three cattle, referencing earlier studies from 1933 that noted *Babesia bovis* as the most commonly confirmed piroplasm at the Skopje slaughterhouse. They also reported the first incidence of cattle anaplasmosis, in October 1936 in Skopje County, with the affected ox presenting with fever and lethargy. *Anaplasma marginale* was discovered in the stained blood smear, but no other piroplasms were present. Mlinac et al. [19] offered a full description of piroplasms infecting animals, thus other authors relied on assumed names of haemoparasites that were later reported in review reports. *Theileria mutans, Theileria dispar, Babesiella bovis, Babesiella maior* n. sp., *Babesiella berbera, Piroplasma bigeminum* and *A. marginale* all occur in cattle. *Theileria ovis* and *Babesiella ovis* were discovered in sheep, while *Babesiella hirci* was found in goats. *Piroplasma caballi* and *Nutallia equi* were found in horses and mules, whereas *Piroplasma canis* was discovered in a dog. In 1935, Petrović [21] provided thorough descriptions of piroplasms in horses.

In 1938, Štrlek [22] examined piroplasms in diseased cattle (*n* = 217), sheep (*n* = 3) and horses (*n* = 7) from the veterinary section of the Skopje Hygiene Institute. It is worth noting that *T. mutans* was identified in 139 blood smears from cattle in Banja Luka (now Bosnia and Herzegovina). In 1939, Horvatić [23] reported from the same hospital three horses (*P. caballi* and *N. equi*), nine sheep (*B. ovis* and *T. ovis*) and 73 cattle (*B. bovis, B. berbera, B. maior* n. sp., *Babesia bigemina, T. mutans, T. dispar* and *A. marginale*) that were infected. He emphasised that cow theileriosis appears to occur primarily around April and May, based on his observations from 1937 to 1939.

In 1929, in Vojvodina, Ranitović [24] described sheep piroplasmosis, which had been happening for years, affecting > 50% of sheep, especially young animals. In 1937, Ćosić [25] reported cattle experiencing a severe death rate of 50–60% owing to piroplasmosis in Serbia's Djerdap mountain and Danub river region (srez ključki). In total, 600 of the region's 8000 cattle were infected. In stained blood smears from the Skopje Hygiene Department, *T. mutans* was identified as the causal agent. During the same time period, data from continental Croatia revealed the existence of piropalsmosis but without a significant influence on animal productivity. Further research conducted by the same author in 1921 proved piroplasmosis in cattle, horses and dogs from the same Croatian littoral region [25]. Surprisingly, statistics from Croatia were based mostly on the results of necropsied animals at the Faculty of Veterinary Medicine in Zagreb. In 1929 piroplasmosis was found in two of 65 necropsied horses from Zagreb County [26]. Piroplasmosis was verified in five horses out of 1169 necropsied horses between 1927 and 1938, accounting for 0.4% of horses in the Zagreb area [27]. During the same time period, piroplasmosis was found by Winterhalter [28] in 23 of the 476 cattle included in his study (4.8%), all of which came from the region near to the Sava River, notably Posavina. Cattle were discovered to be infected with *Piroplasma bovis*, whereas horses were found to be infected with *Piroplasma equi*, based on examination of the merozoites detected in the blood of these animals. At the same time, one case of piroplasmosis was reported in sheep [29, 30]. Rajčević and Butozan [31] claimed to be the first to report a description of piroplasmosis in 12 Croatian cattle. These authors' account of how veterinarians in north-western Croatia were frequently confronted with comparable infections in cattle and faced treatment challenges are particularly noteworthy.

The presence of *Babesia canis* was proven in 1939 through the detection of merozoites in stained blood smears from three dogs exhibiting classic indications of piroplasmosis, such as high temperature, lethargy, splenomegaly, anaemia and icterus [32]. The detection of *Dermacentor reticulatus* ticks on the dogs led the author to associate these ticks with *B. canis* transmission. All dogs were successfully treated with Acarpin.

In 1941 Boko [33] claimed that the Dalmatia region had experienced rare incidences of piroplasmosis prior to 1940, with an increasing number of sick cattle, horses, sheep, and goats being observed around Sinj in 1940. Between May and October of that year, 10% of animals tested positive with ‘*Babesiella bovis*’ on stained blood smears at Knin County’s abattoir. Based on the postmortem findings, the authors concluded that the diseases had a chronic rather than acute course. In the same counties, sheep typically exhibited a peracute or acute course of illness. It is notable that the authors explicitly state that it was impossible to establish the location of infection as the sheep spent nearly 6 months grazing on Bosnian pastures. The earliest examples of *P. caballi* infection in horses were military animals that died with classic acute clinical indications of babesiosis in the Knin area, followed by horses in communities nearby. The causal agent was identified using a Giemsa-stained blood smear.

In those countries that existed in the nineteenth and twentieth centuries in the WBs, ante-mortem and post-mortem examinations were performed at veterinary faculties and/or veterinary (hygiene) institutes, and the causal agents of piroplasmosis were frequently documented in their annual reports. The data presented indicate that piroplasmosis was indeed prevalent throughout the entire region of the WBs, having varying degrees of impact on animal health. The collaboration among experts and scientists has proven to be essential for the extensive data from which we derived data to cover all parts of the WBs, regardless of the existence of additional published data. In the following sections, we provide a comprehensive overview of current knowledge on non-zoonotic TBPs identified in both ticks and animals in the WB countries.

## Albania

Epidemiological data on the prevalence and distribution of non-zoonotic TBPs in ticks, companion animals and livestock in Albania remain scarce, with only sporadic studies providing limited insights into their presence in these populations. Pathogens such as *Anaplasma/Ehrlichia* spp., *Babesia* spp. and *Theileria* spp. have been identified in ticks. In dogs, studies have detected *Babesia canis*, *Ehrlichia canis*, *Hepatozoon* spp. and *Mycoplasma haemocanis*, among others. In livestock, *Anaplasma* spp., *Babesia* spp. and *Theileria* spp. have been reported. No data are available on wild animals, leaving a significant gap in our understanding of the circulation of TBPs in this important reservoirs.

### Ticks

The first comprehensive epidemiological survey on tick-borne bacteria in Albania was carried out by Christova et al. [34] in 2003, who examined 90 ticks from the species *Rhipicephalus bursa*, *Rhipicephalus sanguineus* sensu lato (*Rh. sanguineus* s.l.) and *Hyalomma marginatum* collected from cattle in five localities across northern and central Albania. These ticks were screened for *Anaplasma* and *Ehrlichia* species using PCR and reverse line blot hybridisation (RLBH). The results indicated that 12.5% of *Rh. bursa* and 14.3% of *Rh. sanguineus* s.l. ticks were positive for *Anaplasma/Ehrlichia* by RLBH, while *E. canis* was detected in 3.6% of *Rh. sanguineus* s.l. ticks by PCR (Table 1). Additional research into TBPs was conducted by screening ectoparasites from stray dogs in Tirana for selected agents using PCR [35]. This study highlights the significance of *Rh. sanguineus* s.l. and *Ixodes ricinus* ticks as vectors for a range of pathogens, although *E. canis* was not detected. Koleci et al. [36] conducted the first study on piroplasms in Albania, focusing on ticks collected from goats. Using conventional PCR and sequencing, these authors examined ticks from 17 locations across 15 counties between April and June 2011 and in May 2012. Sequencing of the 18S ribosomal RNA (rRNA) gene fragments revealed the presence of *Theileria ovis* in three flocks, *Babesia ovis* in one flock and *Theileria sergenti* in one flock (Table 1).

**Table 1:** Non-zoonotic tick-borne pathogens in ticks in the Western Balkans

Country	Ixodid tick species^a^	Source	Pathogen^b^	Prevalence (%)	Method^c^	Reference
Albania	*Rh. sanguineus* s.l	Cattle	*Anaplasma/Ehrlichia*	14.3	PCR	[34]
		Cattle	*E. canis*	3.6	PCR	[34]
	*Rh. bursa*	Cattle	*Anaplasma/Ehrlichia*	12.5	PCR	[34]
	*Rh. bursa*	Goats	*T. ovis*	3 flocks	PCR	[36]
		Goats	*B. ovis*	1 flock	PCR	[36]
		Goats	*T. sergenti*	1 flock	PCR	[36]
	*Rh. turanicus*	Goats	*T. ovis*	3 flocks	PCR	[36]
		Goats	*B. ovis*	1 flock	PCR	[36]
		Goats	*T. sergenti*	1 flock	PCR	[36]
Bosnia and Herzegovina	*D. reticulatus*	Dogs	*Babesia* spp.	39	Multiplex PCR	[48]
		Dogs	*Anaplasma* spp.	4.8	Multiplex PCR	[48]
		Cats	*Babesia* spp.	4.8	Multiplex PCR	[48]
	*I. ricinus*	Dogs	*Babesia* spp.	16	Multiplex PCR	[49]
		Cattle	*Babesia* spp.	17.1	Multiplex PCR	[49]
Croatia	*D. reticulatus*	Dog	*H. canis*	1/3	PCR-Seq	[68]
		Red fox	*H. canis*^d^	3/9	PCR-Seq	[68]
	*I. hexagonus*	Red fox	*H. canis*^d^	4/13	PCR-Seq	[68]
		Red fox	*H. canis*^d^	2/14	PCR-Seq	[68]
		Red fox	*Hepatozoon* sp.^d^	1/13	PCR-Seq	[68]
	*I. ricinus*	Dog	*Hepatozoon* sp.	2/20	PCR-Seq	[68]
		Horse	*H. canis*	1/1	PCR-Seq	[68]
		Red fox	*H. canis*^d^	6/19	PCR-Seq	[68]
		Vegetation	*Hepatozoon* sp./*H. martis*	1/53	PCR-Seq	[68]
	*I. canisuga*	Red fox	*H. canis*^d^	3/29	PCR-Seq	[68]
		Red fox	*Hepatozoon* sp.^d^	1/29	PCR-Seq	[68]
		Red fox	*H. canis*^d^	14/24	PCR-Seq	[68]
	*I. ventalloi*	Red fox	*Hepatozoon* sp.^d^	1/2	PCR-Seq	[68]
	*Rh. turanicus*	Cat	*H. felis*	2/3	PCR-Seq	[68]
	*Rh. sanguineus* s.l	Dog	*H. canis*	1/13	PCR-Seq	[68]
	*D. reticulatus*	Vegetation	*B. canis*	77	PCR-Seq	[69]
	*Rh. turanicus*	Sheep	*T. ovis*	1/1	PCR-Seq	[70]
	*Rh. bursa*	Sheep	*T. ovis*	1/2	PCR-Seq	[70]
Serbia	*D. reticulatus*	Dog	*B. canis*	46.4	Blood smear	[105]
	*D. marginatus*	Dog	*B. canis*	18.7	Blood smear	[105]
	*Rh. sanguineus* s.l	Dog	*B. canis*	66.1	Blood smear	[105]
	*D. reticulatus*	Vegetation	*B. canis*	21.5	PCR	[106]
	*H. concinna*	Vegetation	*B. canis*	8.5	PCR	[106]
	*D. reticulatus*	Vegetation	*B. canis*	11/53	PCR, Sequencing	[107]
	*H. concinna*	Vegetation	*B. canis*	3/35	PCR, Sequencing	[107]
		Vegetation	*A. ovis*	20	PCR, Sequencing	[107]
	*H. punctata*	Vegetation	*A. ovis*	50	PCR, Sequencing	[107]
	*I. ricinus*	Vegetation	*A. ovis*	29.6	PCR, Sequencing	[107]
	*D. reticulatus*	Dog	*B. canis*	33.3	PCR, Sequencing	[108]
	*I. ricinus*	Dog	*H. canis*	8.4	PCR, Sequencing	[108]
	*I. ricinus*	Golden jackals	*B. canis*	6/118	PCR, Sequencing	[109]
	*D. reticulatus*	Golden jackals	*B. canis*	8/118	PCR, Sequencing	[109]
		Golden jackals	*A. marginale*	6.4	PCR, Sequencing	[109]
	*Rh. sanguineus* s.l	Dog	*B. gibsoni*	4.1	PCR–RFLP	[110]
		Dog	*B. canis*	12.9	PCR–RFLP	[110]
	*D. reticulatus*	Dog	*B. canis*	44.4	PCR–RFLP	[110]
	*I. ricinus*	Dog	*B. canis*	11.1	PCR–RFLP	[110]
	*Rh. sanguineus* s.l	Dog	*H. canis*	1/4	PCR, Sequencing	[111]

* s.l.* Sensu lato

^a^D., *Dermacentor;* H.,* Hyalomma*; I.,* Ixodes*; Rh.,* Rhipicephalus*

^b^B.,* Babesia*; E.,* Ehrlichia*; H.,* Hepatozoon*; T., *Theileria*

^c^PCR-RFLP, Restriction fragment length polymorphism-PCR; PCR-Seq, PCR followed by DNA sequencing

^d^*Hepatozoon* in ticks collected from negative foxes

### Companion animals

The first report on canine babesiosis in Albania was published in 2006, describing the microscopic identification of *B. canis* in Giemsa-stained blood smears of 23 of 101 dogs from Tirana that were tested in the period from July 2003 to July 2004 [37]. In the same year, Bizhga et al. [38] reported the diagnosis of ehrlichiosis in three dogs based on their examination of Giemsa-stained blood smears. Later studies confirmed the presence of *B. canis* and *Babesia vogeli* DNA in the blood of dogs from Albania and reported an approximately 10% prevalence of anti-*B. canis* antibodies, identified using the indirect fluorescence antibody test (IFAT) [39]. However, it should be taken into consideration that the *B. canis* IFAT may also detect anti-*B. vogeli* antibodies via cross-reactivity with the *B. canis* antigen [40]. In addition to these first records of canine babesiosis in Albania, in 2006, based on the results of their study, Dhamo et al. [37] reported an inverse association between the prevalence of infection and the age of the dogs, positive cases recorded more frequently in spring than in summer and autumn and most cases in dogs with outdoor access.

A subsequent study by Hamel et al. [41] included 30 clinically healthy dogs from suburban areas of Tirana which were screened for *B. canis*, *Hepatozoon* spp. and *E. canis* using both direct and indirect methods. Antibodies or pathogens were found in 67% (20/30) of the dogs. Notably, 63% (19/30) of the dogs had antibodies against *B. canis*, *E. canis*, *B. vogeli*, *Hepatozoon* spp. and *E. canis* were identified in 43% (13/30) of the dogs through blood smear, PCR, or enzyme-linked immunosorbent assay (ELISA) (Table 2). In a later study, Hamel et al. [42] screened 602 client-owned dogs that had presented to four small animal clinics between March 2010 and April 2011 in Tirana using Giemsa-stained blood smears, PCR and serological methods to determine the presence/absence of arthropod-borne infections. *Babesia vogeli, Hepatozoon canis, Anaplasma platys, E. canis,* and *M. haemocanis*, were detected by direct methods with prevalence rates ranging from 1 to 9%. Seroprevalence for *Babesia* spp., *Anaplasma* spp., and *E. canis* were 6.6, 24.1, and 20.8%, respectively (Table 2).

**Table 2:** Non-zoonotic tick-borne pathogens affecting companion animals in the Western Balkans

Country	Pathogen^a^	Host species	Prevalence (%)	Method^b^	Reference
Albania	*B. canis*	Dog	23/101	Blood smear	[37]
	*E. canis*	Dog	–	Blood smear	[38]
	*B. canis, B. vogeli*	Dog	10	IFAT	[39]
	*B. c. canis, E. canis*	Dog	63	Blood smear, PCR, ELISA	[41]
	*B. c. vogeli, E. canis, Hepatozoon* spp.,	Dog	43	Blood smear, PCR, ELISA	[41]
	*Babesia* spp.	Dog	6.6	Blood smear, PCR, Serology	[42]
	*Anaplasma* spp.	Dog	24.1	Blood smear, PCR, Serology	[42]
	*E. canis*	Dog	20.8	Blood smear, PCR, Serology	[42]
	*Hepatozoon* spp.	Dog	1	Blood smear	[43]
	*Babesia* spp.	Dog	0.2	Blood smear	[43]
	*B. vogeli*	Dog	0.3	qPCR	[43]
	*Mycoplasma haemocanis*	Dog	8.8	qPCR	[43]
	*A. platys*	Dog	3.3	PCR	[43]
	*E. canis*	Dog	9.5	PCR	[43]
	*Babesia* spp.	Dog	6.6	Serology	[43]
	*E. canis*	Dog	20.8	Serology	[43]
	*Anaplasma* spp.	Dog	24.1	Serology	[43]
Bosnia and Herzegovina	*Babesia* spp.	Dog	27.9	Blood smear	[50]
	*Babesia* spp.	Dog	5.2	Blood smear	[51]
	*Babesia* spp.	Dog	30.6	Blood smear	[52]
	*B. canis*	Dog	82.5–85	Blood smear, PCR-Seq	[53]
	*E. canis/E. ewingii*	Dog	0.2	SNAP 4Dx	[54]
	*A. platys*	Dog	0.2	qPCR	[55]
	*B. vogeli*	Dog	0.2	qPCR	[55]
	*Apicomplexa*	Dog	35	qPCR	[55]
	*Hepatozoon* spp.	Dog	26	qPCR	[55]
Croatia	*B. canis*	Dog (symptomatic)	8 cases	Blood smear, PCR-Seq	[71]
	*B. canis*	Dog (symptomatic)	96.3	Blood smear, PCR-Seq	[72]
		Dog (asymptomatic)	2.4	PCR-Seq	[72]
	*B. vogeli*	Dog (symptomatic)	1.3	Blood smear, PCR-Seq	[72]
		Dog (asymptomatic)	0.2	PCR-Seq	[72]
	*T. equi*	Dog (symptomatic)	1.3	Blood smear, PCR-Seq	[72]
	*B. caballi*	Dog (symptomatic)	1.3	Blood smear, PCR-Seq	[72]
	*B. gibsoni*	Dog (asymptomatic)	0.7	PCR-Seq	[72]
	*B. vulpes*	Dog (asymptomatic)	0.1	PCR-Seq	[72]
	*B. canis*	Dog (symptomatic)	28/29	PCR-Seq	[73]
	*B. canis*	Dog	11/19 cases	PCR-Seq	[74]
	*T. capreoli*	Dog	1/19 cases	PCR-Seq	[74]
	*B. canis*	Dog	13/14 cases	PCR-Seq	[75]
	*B. canis*	Dog	8/8 cases	PCR-Seq	[75]
	*H. canis*	Dog (asymptomatic)	11.5	PCR-Seq	[76]
	*Hepatozoon* sp.	Dog (asymptomatic)	0.2	PCR-Seq	[76]
	*A. platys/B. vogeli*	Dog	1, case report	IFA, SNAP 4Dx test, RT-PCR-Seq	[77]
	*B. canis*	Dog	20	IFA	[78]
	*Anaplasma* spp.	Dog	6.2	SNAP 4Dx test	[78]
	*E. canis*	Dog	0.5	SNAP 4Dx test	[78]
	*Anaplasma* spp.	Dog	3.2–5.4	SNAP 4Dx test	[79]
	*E. canis*	Dog	0.0–0.6	SNAP 4Dx test	[79]
	*A. platys*	Dog	2.5	PCR-Seq	[80]
Montenegro	*E. canis*	Dog	19.3	IFAT	[89]
	*E. canis*	Dog	10 cases	IFAT	[92]
	*Babesia* sp.	Dog	6 cases	Blood smear	[92]
	*B. canis*	Dog	1 case	Blood smear	[92]
	*B. gibsoni*	Dog	1 case	Blood smear	[92]
Serbia	*B. canis*	Dogs	58 cases	PCR–RFLP, Sequencing	[116]
	*B. gibsoni*	Dogs	3.3	PCR–RFLP, Sequencing	[116]
	*A. platys*	Dogs	0.9	ELISA	[117]
	*B. canis*	Dogs	13.5	PCR	[117]
	*B. gibsoni*	Dogs	2.7	PCR	[117]
	*B. vogeli*	Dogs	0	PCR	[117]
	*B. canis*	Dogs	51.4	IFAT	[117]
	*B. gibsoni*	Dogs	12.6	IFAT	[117]
	*B. vogeli*	Dogs	52.3	IFAT	[117]
	*B. vulpes*	Dogs	10.1	PCR, Sequencing	[118]
	*B. gibsoni*	Dogs	5.7	PCR, Sequencing	[118]
	*B. vogeli*	Dogs	1.9	PCR, Sequencing	[118]
	*B. caballi*	Dogs	1.9	PCR, Sequencing	[118]
	*H. canis*	Dogs	0.6	PCR, Sequencing	[118]
	*B. canis*	Dogs	26.2	IFAT	[119]
	*B. canis*	Hunting dogs	32.8	IFAT	[120]
	*A. platys*	Dog	1 case	PCR, Sequencing	[123]
	*H. canis*	Dog	1 case	PCR, Sequencing	[124]

* s.l.* Sensu lato

^a^A., *Anaplasma*; B.,* Babesia*; D.,Dermacentor; E.,* Ehrlichia*; H.,* Hyalomma*; T., *Theileria*

^b^ELISA, Enzyme-linked immunosorbent assay; IFAT, indirect immunofluorescence test; PCR-RFLP, restriction fragment length polymorphism-PCR; PCR-Seq, PCR followed by DNA sequencing; qPCR, real-time PCR; RT-PCE, reverse transcription PCR

The current study represents the first molecular evidence of *A. platys, E. canis,* and *M. haemocanis* in Albania [43]. More recently, haematological and clinical findings in dogs from Albania with microscopically confirmed *Babesia* infection were reported [44, 45]. In endemic areas, there is a strong association beween the *Babesia* species that is transmitted and the tick vector present in the environment.

### Livestock

Petrovec et al. [46] conducted a study from May to July 2000 on internal organs collected after evisceration of 203 slaughtered calves, sheep and goats across the district of Shkodra in the north of Albania using a molecular approach. Three different *Anaplasma* species (*A. marginale, Anaplasma centrale* and *A. ovis*) were detected, with a prevalence of 48% (35/73) in sheep, 44% (30/68) in goats and 22.6% (14/62) in calves. One sample (amplified from sheep) showed the highest homology (99.1%) to *Ehrlichia* sp. strain Ommatjene. Zalla et al. [47] performed a study on 186 cattle in north central Albania in 2008 using a haematological assay. Of the total number of samples testing positive for infection, 2.1% were infected with *B. bigemina*, 3.2% were infected with both *B. bigemina* and *B. bovis* and 1% had cross infection (*Babesia* spp. and *Anaplasma* spp.) (Table 3).

**Table 3:** Non-zoonotic tick-borne pathogens affecting livestock in the Western Balkans

Country	Pathogen^a^	Host species	Prevalence (%)	Method^b^	Reference
Albania	*Anaplasma* spp.	Sheep	48 (35/73)	PCR	[46]
	*Anaplasma* spp.	Goat	44 (30/68)	PCR	[46]
	*Anaplasma* spp.	Calf	2.6 (14/62)	PCR	[46]
	*B. bigemina*	Cattle	2.1	Blood smear	[47]
	*B. bigemina/B. bovis*	Cattle	3.2	Blood smear	[47]
	*Babesia* spp./ *Anaplasma* spp.	Cattle	1	Blood smear	[47]
Bosnia and Herzegovina	*B. caballi*	Horse	4.2	PCR-Seq	[58]
	*B. ovis*	Sheep	36.4	PCR-Seq	[59]
	*T. orientalis*	Cattle	43	PCR-Seq	[60]
	*A. ovis*	Sheep	46.9	PCR	[61]
	*A. ovis/B. ovis*	Sheep	63.3	PCR	[61]
Croatia	*T. ovis*	Sheep	50–71	PCR-Seq	[70]
	*Theileria* sp. OT3	Sheep	14–40	PCR-Seq	[70]
	*A. ovis*	Ram	1 clinical case	Blood smear, PCR-Seq	[81]
	*A. marginale*	Cattle	5 cows	Spleen imprint, PCR-Seq	[82]
	*A. bovis*	Cattle	3 cows	PCR-Seq	[82]
	*T. orientalis*	Cattle	3 cows	PCR-Seq	[82]
	*B. caballi*	Horse	13/14 clinical cases	PCR-Seq	[85]
	*T. equi*	Horse	1/14 clinical cases	PCR-Seq	[85]
	*T. equi/B.caballi*	Horse	24.7	ELISA	[85]
Montenegro	*T. equi*	Horse	22.5	PCR	[58]
	*B. caballi*	Horse	2.1	PCR	[58]
	*Babesia* sp.	Sheep	8 cases	Blood smear	[92]
	*Babesia* sp.	Cattle	4 cases	Blood smear	[92]
	*Babesia* sp.	Goat	1 case	Blood smear	[92]
	*A. marginale*	Sheep	4 cases	Blood smear	[92]
North Macedonia	*B. ovis*	Sheep	N/A	N/A	[17]
	*T. ovis*	Sheep	N/A	N/A	[21]
	*B. caballi*	Horse	N/A	N/A	[93]
	*T. hirci*	Goat	N/A	Blood smear	[94]
	*Theileria* spp.	Cattle	N/A	N/A	[96]
	*B. ovis*	Sheep	N/A	N/A	[97, 98]
	*B. ovis*	Sheep	N/A	N/A	[99]
	*B. ovis*	Sheep	N/A	N/A	[101]
	*B. ovis*	Goat	N/A	N/A	[102]
	*B. ovis*	Goat	20.7 adults; 21.9 juveniles	Blood smear	[103]
Serbia	*T. equi*	Horses	27.7	PCR, Sequencing	[58]
	*A. marginale*	Cattle	11.9	Light microscopy	[125]
	*T. annulata*	Cattle	1.4	Light microscopy	[125]
	*B. bigemina*	Cattle	3.6	Light microscopy	[125]
	*B. bovis*	Cattle	5.7	Light microscopy	[125]
	*T. equi*	Donkeys	50	PCR, Sequencing	[128]
	*B. caballi*	Donkeys	0	PCR	[128]
	*Theileria* spp.	Cattle	3.7	PCR, Sequencing	[129]

^a^A., *Anaplasma*; B.,* Babesia*; T., *Theileria*

^b^ELISA, Enzyme-linked immunosorbent assay; N/A, not available; PCR-Seq, PCR followed by DNA sequencing

## Bosnia and Herzegovina

Comprehensive epidemiological data on the prevalence and distribution of non-zoonotic TBPs in ticks, companion animals, livestock and wild animals are scarce in Bosnia and Herzegovina. Overall, such research has been sporadic, with occasional reports confirming established prevalence in animals within the mentioned groups. Recent research on pathogens identified in ticks in Bosnia and Herzegovina has revealed the presence of *Babesia* spp. and *Anaplasma* spp. Studies on companion animals have primarily focused on dogs, which have been shown to harbor a range of pathogens, including *B. canis*, *E. canis*/*Ehrlichia ewingii*, *A. platys*, *B. vogeli*, Apicomplexa and *Hepatozoon* spp. In livestock, documented pathogens include *Babesia caballi*, *Babesia ovis*, *Theileria orientalis* and *Anaplasma ovis,* while data on non-zoonotic tick-borne pathogens in goats remain unavailable. Among wild animals, investigations have been conducted on foxes, wild cats, martens and wolves, resulting in the identification of pathogens such as *B. canis, Babesia vulpes, H. canis, Hepatozoon silvestris, Hepatozoon felis, Cytauxzoon* sp. and *Hepatozoon* sp., reflecting a diverse range of pathogen reservoirs.

### Ticks

Recent studies on the molecular detection of pathogens in ticks have recently been conducted, specifically in the species *I. ricinus*, *Ixodes hexagonus*, *Ixodes canisuga* and *D. reticulatus* [48, 49]. In one study on *D. reticulatus* collected from dogs, cats and sheep, the presence of *Babesia* spp. and *Anaplasma* spp. was confirmed, with frequencies ranging from 4.8% to 51.2% [48]. Among the *Ixodes* species, only *I. ricinus* collected from dogs, cats, cattle, sheep and goats showed the presence of *Babesia* spp., with frequencies ranging from 4.8% to 17.1% [49] (Table 1). These results highlighted the expansion of the host range and distribution of ticks and that this expansion may have significant implications for the epidemiology of TBDs in Bosnia and Herzegovina.

### Companion animals

The initial investigation of non-zoonotic TBPs in companion animals in Bosnia and Herzegovina was conducted by Omeragic et al. [50]. These authors examined the peripheral blood smears of 44 dogs in the Sarajevo area that exhibited clinical symptoms of babesiosis, and identified *Babesia* spp. in 12 dogs (27.9%). In a subsequent study conducted in Tuzla by Omeragic et al. [51], peripheral blood samples from 134 dogs were examined, revealing the presence of *B. canis* in seven dogs (5.2%), and an investigation of blood smears from the peripheral blood of 183 dogs in the municipality of Teslić conducted by Majkić et al. [52] confirmed a slightly higher prevalence of *Babesia* spp. infection (30.6%) (Table 2). The peak incidence was in May, totaling 20 infections (35.7%), followed by June (*n* = 10, 28.5%), July (*n* = 9, 16%), August (*n* = 7, 12.5%), September (*n* = 5, 8.9%), April (*n* = 3, 5.3%) and March (*n* = 2, 3.5%), which highlighted the seasonality of disease occurrence.

The initial molecular investigation of *B. canis* in dogs from Sarajevo, conducted by Ćoralić et al. [53], confirmed a notably high prevalence of autochthonous babesiosis in naturally infected dogs exhibiting symptoms. Among 80 dogs with clinical signs of babesiosis, *Babesia* was identified in the blood smears of 82.5% of the dogs. Molecular (PCR) techniques, applied to all parasitologically positive and two negative samples, confirmed infection with *Babesia* species in 85% of instances. In addition, sequence analysis demonstrated 100% homology with *B. canis* sequences (Table 2). A more comprehensive investigation of Anaplasmataceae was conducted in 2017, utilising the SNAP 4Dx Plus test and real-time PCR (qPCR). A total of 903 blood samples from stray dogs were analysed for the presence of antibodies against *E. canis/E. ewingii*. Antibodies were detected in 187 samples (20.7%), with two dogs exhibiting antibodies against *E. canis*/*E. ewingii* and one dog exhibiting antibodies against both. Among the 187 seropositive dogs analysed using qPCR, 48 (25.7%) tested positive for Anaplasmataceae.. Two samples positive for Anaplasmataceae did not show the presence of the mentioned species in species-specific PCR tests [54] (Table 2).

In a recent comprehensive study on vector-borne pathogens (VBPs) in companion animals, Colella et al. [55] collected blood samples from 408 domestic dogs and tested them using a microfluidic real-time PCR assay for 43 different pathogens. The study revealed the presence of individual and mixed infections. *Anaplasma platys* was confirmed in one dog in the Mostar region (0.2%) and *B. vogeli* was identified in two dogs in Sarajevo and one dog in Bihać (0.7%). Apicomplexa was the predominant finding in 141 dogs (35%), followed by *Hepatozoon* spp. in 107 dogs (26%).

### Livestock

In a study conducted in 1936, Kozinc [56] obtained initial data on the pathogenic impact of ticks on sheep, goats and cattle in the territory of Bosnia and Herzegovina. This author described the occurrence of a phenomenon known as 'leđanica' in November and December in the present-day municipality of Konjic, specifically in the villages of Ljuta, Jošanica, Spiljani and Bijela, attributing the emergence of 'leđanica' to the invasion of *I. ricinus*. The first investigation into TBPs was conducted by Papić [57] in 1976 in Bugojno municipality, where he observed babesiosis in the spring and summer, reporting that its presence was influenced by ecological conditions favorable for tick development, which in turn spurred increased interest in monitoring the disease. Papić's study in 1976 [57] revealed a high prevalence (69.2%) of bovine babesiosis in Bosnia and Herzegovina, with the author suggesting that, at least during that period, the disease was endemic in the central region of the country.

In 2016, Davitkov et al. [58] conducted the first study on equine babesiosis. Blood samples were collected from 24 horses, and the presence of *B. caballi* was confirmed in one horse (4.2%) using PCR and sequencing. Research on babesiosis in sheep in Bosnia and Herzegovina was conducted in 2022 by Stevanović et al. [59]. These authors collected blood samples from a total of 192 clinically asymptomatic (*n* = 116) and clinically suspected sheep (*n* = 76) from 53 flocks in the Podrinje and Eastern Herzegovina regions. Molecular confirmation of *B. ovis* was conducted using PCR. Of the 192 tested sheep, *B. ovis* was confirmed in 70 (36.4%) of them [59]; specifically, *B. ovis* was confirmed in 11.2% (13/116) of asymptomatic sheep, while in clinically suspected cases, the positivity rate was 75% (57/76). The majority of clinical cases of malignant ovine babesiosis were confirmed in the Rudo epidemiological unit (78.7%) within the Podrinje region, indicating a typical seasonal pattern of disease occurrence and an endemic focus. Most babesiosis cases were diagnosed in July (*n* = 37), followed by June (*n* = 17), August (*n* = 2) and May (*n* = 1) (Table 3).

In a recent study on TBPs in cattle (2023), Stevanović and Radalj [60] confirmed the presence of DNA fragments specific to *Babesia/Theileria* in 13 out of 30 examined cattle (43%). At the farm level, PCR-positive animals were identified on 60% of surveyed farms, with 100% positivity observed in cattle from three farms with a history of babesiosis cases. Additionally, sequence analysis confirmed the presence of *T. orientalis*. Also, in the latest study by Stevanović et al. [61] on TBPs in sheep, the presence of *A. ovis* was confirmed in 38 out of 81 (46.9%) sheep from the Podrinje and Herzegovina regions, while mixed infections with *B. ovis* and *A. ovis* were observed in 63.3% of cases. These studies highlighted the emergence of new genotypes and high genetic variability of *A. ovis*, which were not associated with geographic origin, tick-borne infection status or sheep breeding practices in Podrinje and Herzegovina (Table 3).

### Wild animals

The first investigation of *B. canis*, *B. vulpes* (previously known as *Babesia* cf. *microti*) and *H. canis* in foxes in Bosnia and Herzegovina was conducted in 2015 by Hodžić et al. [62]. Spleen samples from 119 foxes were collected in 29 municipalities across six different regions during the hunting season. DNA of *B. canis*, *B. vulpes* and *H. canis* was identified in one (0.8%), 38 (31.9%) and 46 (38.6%) spleen samples, respectively. Additionally, the study confirmed the existence of mixed infections in foxes, with co-infections of *B. vulpes* and *H. canis* identified in 11 foxes (9.2%), while one fox carried all three pathogens (0.8%). The authors used molecular methods to confirm *B. vulpes* in foxes across all six investigated regions in Bosnia and Herzegovina, with the highest frequency (66.6%) recorded in Herzegovina (Table 4).

**Table 4:** Non-zoonotic tick-borne pathogens affecting wild animals in the Western Balkans

Country	Pathogen^a^	Host species	Prevalence (%)	Method^b^	Reference
Bosnia and Herzegovina	*B. canis*	Red fox	0.8	PCR-Seq	[62]
	*B. vulpes*	Red fox	31.9	PCR-Seq	[62]
	*H. canis*	Red fox	38.6	PCR-Seq	[62]
	*H. silvestris* sp. nov	Wild cat	56	PCR-Seq	[63]
	*H. felis*	Wild cat	11	PCR-Seq	[63]
	*Babesia* sp.	Wild cat	6	PCR	[64]
	*Cytauxzoon* sp.	Wild cat	56	PCR	[64]
	*H. silvestris*	Wild cat	22	PCR	[64]
	*H. felis*	Wild cat	33	PCR	[64]
	*Hepatozoon* sp.	Wild cat	22	PCR	[64]
	*Corynebacterium europaeus*	Wild felids	N/A	PCR	[65]
	*H. martis* n. sp.	Marten	64	PCR-Seq	[66]
	*H. canis*	Red fox	100	PCR-Seq	[67]
	*H. canis*	Wolf	100	PCR-Seq	[68]
Croatia	*H. martis*	Stone marten	63.6	PCR-Seq	[66]
	*H. canis*	Golden jackal	80.8	PCR-Seq	[68]
	*H. canis*	Grey wolf	54.2	PCR-Seq	[68]
	*H. canis*	Badger	7.8	PCR-Seq	[68]
	*H. martis*	Badger	1.6	PCR-Seq	[68]
	*Hepatozoon* sp.	Bank vole	81.8	PCR-Seq	[68]
	*Hepatozoon* sp.	Yellow-necked mouse	2.7	PCR-Seq	[68]
	*Hepatozoon* sp.	Wood mouse	10.4	PCR-Seq	[68]
	*H. ayorgbor*	Yellow-necked mouse	5.4	PCR-Seq	[68]
	*H. ayorgbor*	Wood mouse	4.2	PCR-Seq	[68]
	*H. sciuri*	European hedgehog	100	PCR-Seq	[68]
	*H. canis*	Red fox	23	PCR-Seq	[86]
	*Hepatozoon* sp.	Red fox	1	PCR-Seq	[86]
	*B. vulpes*	Red fox	5.2	PCR-Seq	[86]
	*Theileria* sp.	Red fox	1	PCR-Seq	[86]
	*B. canis*	Grey wolf	5.5	PCR-Seq	[87]
	*T. capreoli*	Grey wolf	13.9	PCR-Seq	[87]
	*Babesia sp.*	Red deer	2.9	PCR-Seq	[88]
	*B. divergens/capreoli*	Red deer	0.9	PCR-Seq	[88]
	*B. divergens/capreoli*	Roe deer	18.3	PCR-Seq	[88]
	*B. crassa*	Roe deer	2	PCR-Seq	[88]
	*B. venatorum*	Roe deer	2	PCR-Seq	[88]
	*T. capreoli*	Red deer	52.9	PCR-Seq	[88]
	*T. capreoli*	Roe deer	57.1	PCR-Seq	[88]
	*T. capreoli*	Fallow deer	100	PCR-Seq	[88]
	*T. ovis*	Roe deer	2	PCR-Seq	[88]
Serbia	*B. canis*	Golden jackal	4.2	PCR, Sequencing	[108]
	*B. canis*	Red fox	0.8	PCR, Sequencing	[130]
	*B. vulpes*	Red fox	28.7	PCR, Sequencing	[130]
	*H. canis*	Red fox	61.2	PCR, Sequencing	[130]
	*H. canis*	Grey Wolf	57.9	PCR, Sequencing	[132]
	*H. canis*	Yellow-necked mouse	One case	PCR, Sequrencing	[133]

^a^B.,* Babesia*; H.,* Hyalomma*; T., *Theileria*

^b^PCR-Seq, PCR followed by DNA sequencing

*Hepatozoon silvestris* was confirmed in wildcats by Hodžić et al. [63] based on morphological and genetic characteristics. Tissue samples were collected from nine European wildcats in the areas of five municipalities in northwestern (Bihać, Bosanski Petrovac), northern (Odžak), eastern (Goražde) and central (Gornji Vakuf) Bosnia and Herzegovina, where histopathological and molecular analyses were conducted. Histopathological analysis revealed various developmental stages of *Hepatozoon* meronts observed in multiple cross-sections in the heart, lungs, spleen and skeletal muscle tissue in four (44%) out of the nine European wildcats. Additionally, tissues from six animals (67%) tested positive by PCR. *Hepatozoon felis* was identified as the causative agent of infection in one cat (11%), while 18S rRNA sequences from the remaining five cats (56%) were found to be identical but distinct from *H. felis* sequences. In addition, phylogenetic analyses revealed that these sequences formed a strongly supported branch distant from other *Hepatozoon* species, supporting the discovery of a new species, *H. silvestris* sp. nov. (Table 4).

In a subsequent study conducted by Hodžić et al. [64], blood-associated parasites were confirmed in 18 European wildcats using PCR. The presence of five species of apicomplexan parasites belonging to three genera (*Babesia* sp., *Cytauxzoon* sp., *H. silvestris*, *H. felis*, *Hepatozoon* sp.) was established. At least one of these microorganisms was detected in 15 wildcats (83%). *Cytauxzoon* sp. was the most frequently identified pathogen (56%; 10/18), followed by *H. felis* (33%; 3/9), *H. silvestris* (22%; 2/9), *Hepatozoon* sp. (22%; 2/9) and *Babesia* sp. (6%; 1/18). Additional molecular analysis revealed that all *Cytauxzoon* sequences obtained from wild felids in Bosnia and Herzegovina belong to a predominant European haplogroup (EU1). This haplogroup has been identified as a distinct species and formally named *Cytauxzoon europaeus* [65]. Double infections were observed in five animals, while one wildcat carried as many as three different pathogens. Blood, spleen and heart samples were utilised for pathogen detection, with the highest overall positivity rate observed in the blood (100%; 6/6).

The analysis of samples from European martens (*Martes martes*) in Bosnia and Herzegovina and Croatia, as part of a broader study, unveiled a new species of *Hepatozoon* named *Hepatozoon martis* [66]. Collected from various locations in Bosnia and Herzegovina between 2014 and 2017, a total of 10 European martens (9 males and 1 female; 9 adults and 1 cub) were included in the study. The overall prevalence of infection with *H. martis*, detected by PCR in martens from Bosnia and Herzegovina, reached 64% (Table 4).

In 2021, Alić et al. [67] conducted a study on *H. canis* in foxes, with histopathological examination of a red fox cub revealing the presence of *Hepatozoon* spp. meronts in the bone marrow, spleen, lymph nodes and diaphragmatic lung lobes. Additionally, PCR and sequencing confirmed the presence of *H. canis* in the tissues. More recently, Uiterwijk et al. [68] conducted a comprehensive study, testing a larger number of samples from wild mammals across multiple European countries using PCR and sequencing. In 35 samples from wild boars in Bosnia and Herzegovina, no presence of *Hepatozoon* spp. or any other TBPs was established. However, in one wolf sample, the presence of *H. canis* was confirmed [68].

## Croatia

In the Republic of Croatia, most studies have focused on dogs and wild canids, while investigations into the prevalence of non-zoonotic TBPs in ticks remain limited. Nevertheless, ticks have been found to harbor *H. canis, Hepatozoon* sp., *B. canis* and *T. ovis*. Studies on companion animals, primarily dogs, have documented a range of pathogens, including *B. canis*, *B. vogeli*, *Theileria equi*, *B. caballi*, *A. platys*, *E. canis* and *Hepatozoon* sp., with *Babesia gibsoni* and *B. vulpes* reported at relatively lower prevalence. In livestock, investigations have revealed the presence of *T. ovis, T. orientalis, A. ovis, A. marginale* and *T. equi/B. caballi*, while data on non-zoonotic tick-borne pathogens in goats are lacking. Wild animals serve as important reservoirs, with species such as *H. canis, H. martis, B. vulpes, Theileria capreoli* and *Babesia* sp. frequently identified.

### Ticks

So far, only a few studies have investigated the presence of pathogens in ticks. In Zagreb, *B. canis* was found in 77% of pooled *D. reticulatus* ticks from the same location [69]. In southern Croatia, the DNA of* T. ovis* was detected in two ticks, *Rhipicephalus turanicus* and *Rh. bursa*, collected from infected sheep while *Haemaphysalis sulcata* and *Hae. punctata* were found to be negative [70]. In the same study, *Theileria* sp. OT3 was not identified in ticks, despite these ticks having been collected from sheep confirmed to be infected. Uiterwijk et al. [68] tested animals and ticks collected from both the environment and animals for the presence of *Hepatozoon* spp. using PCR and sequencing methods. In the 31 positive (4.1%) ticks, *Hepatozoon* species associated with carnivores were detected, including mostly *H. canis* and, to a lesser extent, *H. martis* and *H. felis*. These authors detected *H. canis* not only in *Rh. sanguineus* s.l., but also in *D. reticulatus*, *I. hexagonus*, *I. ricinus*, *I. canisuga* and *Ixodes ventalloi*, while *H. martis* was present only in questing *I. ricinus* and *H. felis* was present only in *Rh. turanicus* collected from a cat (Table 1).

### Companion animals

**Fig. 1:**
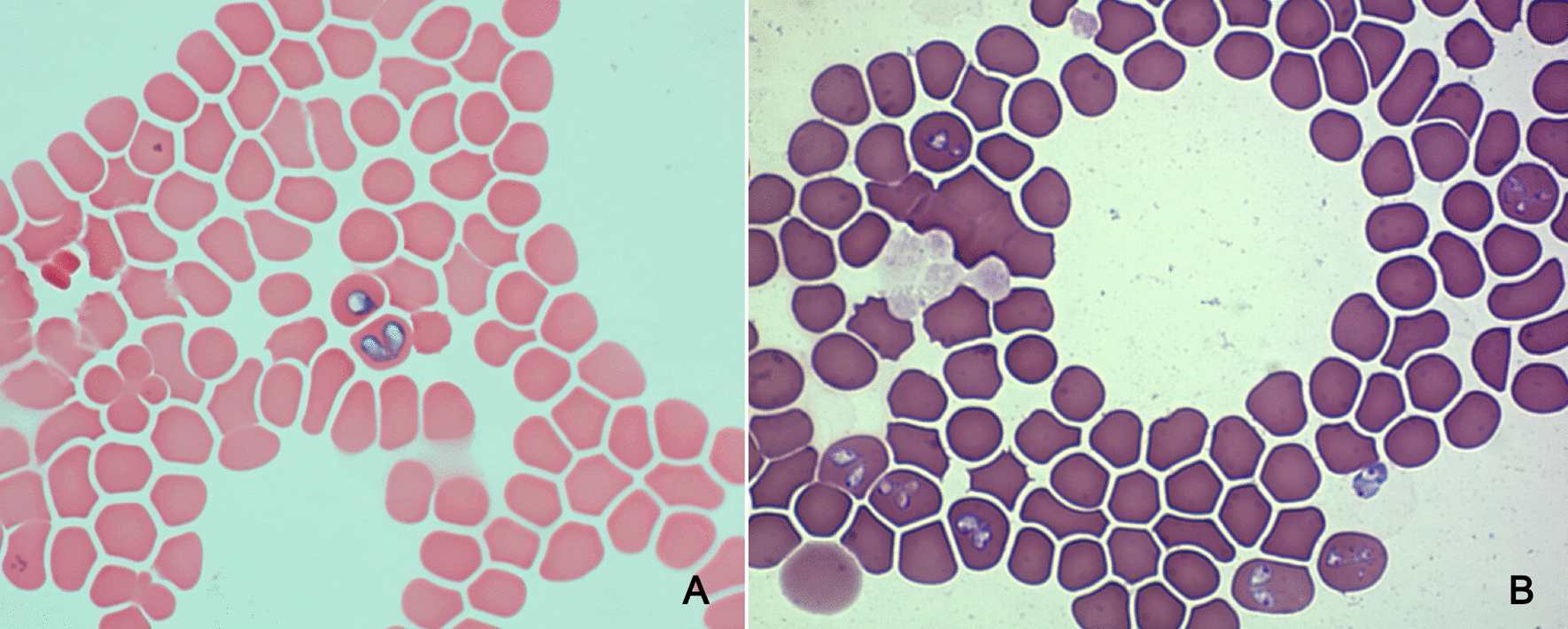
**a**, **b** Giemsa-stained smears with intra-erythrocytic merozoites of *Babesia canis* infection (×1000)

In the first molecular study in Croatia, published in 2002, *B. canis* was detected in eight dogs from the Zagreb region that showed clinical signs of babesiosis, including apathy, fever and anaemia, after sequencing of 18S rRNA [71]. In a large molecular study of 81 dogs that were microscopically positive for babesiosis and 848 randomly selected, apparently healthy dogs, Beck et al. [72] detected six piroplasm species. Sequencing of a portion of the 18S rRNA revealed that *B. canis* was the dominant species, identified in 78 of the symptomatic dogs (96%), followed by single infections (1.3%) with *B. vogeli, B. caballi* and *T. equi*. In a group of randomly selected, apparently healthy dogs, the prevalence was 3.4%, with *B. canis* detected in 20 dogs (2.4%), *Babesia gibsoni* detected in six dogs (0.7%), *B. vogeli* detected in two dogs (0.2%) and *B. vulpes* detected in a single dog (0.1%) (Table 2). This study was the first to provide evidence of *B. vulpes* outside of Spain and the first that recognised *B. gibsoni* in the WB region. In 2010, Brkljačić et al. [73], using the same approach, confirmed the presence of *B. canis* in 28 dogs exhibiting lethargy, anorexia, fever, dark urine and thrombocytopenia, following the detection of merozoites in blood smears (Fig. 1).

In retrospective post-mortem studies on archived, formalin-fixed, paraffin-embedded tissue blocks (FFPEB) from dogs that had died due to a haemolytic crisis, *B. canis* was confirmed in 52.6% (10/19) of the dogs and *T. capreoli* was recorded in the heart tissue of a single dog (5.2%) [74]. In another post-mortem study, *B. canis* was the only species confirmed by sequencing from archived Romanowsky stained cytological slides, in which canine piroplasmosis had been previously identified after microscopic examination [75]. These authors also amplified *B. canis* from the different tissues of 15 dogs that had shown gross findings consistent with haemolytic disease, despite the clearance of merozoites after treatment [75]. Interestingly the highest prevalence was found in the region where *B. canis* had not recorded so far.

In 2009 Vojta et al. [76] performed the first molecular survey to investigate the prevalence of *Hepatozoon* infection in 924 blood samples of apparently healthy dogs from different regions of Croatia. Screening with PCR revealed the presence of *Hepatozoon* DNA in 108 (11.8%) dogs, and sequencing results confirmed the presence of *H. canis* in 106 dogs and *Hepatozoon* sp. in two dogs. The *H. canis* isolates were divided into five groups based on eight commonly mutated nucleotide positions in the partial 18S rRNA gene sequence, (Table 2). In 2012 Dyachenko et al. [77] reported for the first time a dog from Croatia imported to Germany with a lethal infection caused by *A. platys*. The dog developed thrombocytopenia, anaemia and elevated levels of C-reactive protein, with the severity of the condition attributed to co-infection with *B. vogeli*.

Two studies have been performed so far in Croatia with the aim to detect antibodies to TBPs in dogs from different regions. In 2017, Mrljak et al. [78] investigated 435 randomly selected apparently healthy dogs in 13 different locations of Croatia for antibodies to *B. canis* by indirect immunofluorescence using a commercial IFA and commercial point-of-care SNAP®4Dx®Plus. *Babesia canis* was the most prevalent pathogen (20%) while the antibodies to *Anaplasma* spp. were present in 6.2% dogs with a homogeneous geographical distribution throughout the country (Table 2). Antibodies to *E. canis* were present in 0.4% of dogs. In a subsequent study, Jurković et al. [79] conducted large-scale screening of 1433 dogs from the continental and coastal regions that had been categorised by health status. The first group (asymptomatic) included 753 apparently healthy dogs (52.6%, 753/1433); the second group (clinically suspected) comprised 617 dogs (43.1%, 617/1433) that had been presented to private veterinary clinics due to clinical signs and/or haematological abnormalities (anaemia, thrombocytopenia, vomiting, anorexia, pale mucous membranes); the third group (deceased) consisted of 63 dogs (4.4%, 63/1433) with suspected canine VBDs. The screening revealed that the most frequently detected antibodies were those to *Anaplasma* spp. (4.5%). The overall prevalence was the highest in the group of asymptomatic dogs (5.4%) compared to suspected (3.4%) or deceased dogs (3.2%) and was higher in the Continental region than in the Coastal region. Antibodies to *E. canis* were present in 0.6% of dogs (asymptomatic and suspected), but deceased dogs were not seropositive. Interestingly the highest prevalence was noted in the group of asymptomatic dogs (1.4%) from the continental region while the prevalence in the same group from the coastal region was 0.5% [79]. In a study of 1080 blood samples from apparently healthy dogs from the coastal and continental parts of Croatia, Anaplasmataceae DNA was found to be present in 42/1080 (3.8%) dogs using conventional PCR and sequencing of the 16S rRNA gene [80]. Further analysis of the positive samples revealed the presence of *A. platys* (2.5%, 27 dogs) and a *Wolbachia* sp. endosymbiont of *Dirofilaria repens* (1.1%, 12 dogs) (Table 2). The highest prevalence of Anaplasmataceae-positive dogs was identified in the North Adriatic region (10/126; 7.9%) followed by the continental region (11/242; 4.5%) and Dalmatia (21/712; 2.9%). In the same study [80], tissue samples collected from 63 deceased dogs with a history of anaemia and thrombocytopenia were found to be free from infection. All groups were free of *E. canis* DNA despite 838 dogss coming from the coast region where the *Rh. sanguineus* s.l. vector is widespread.

### Livestock

**Fig. 2:**
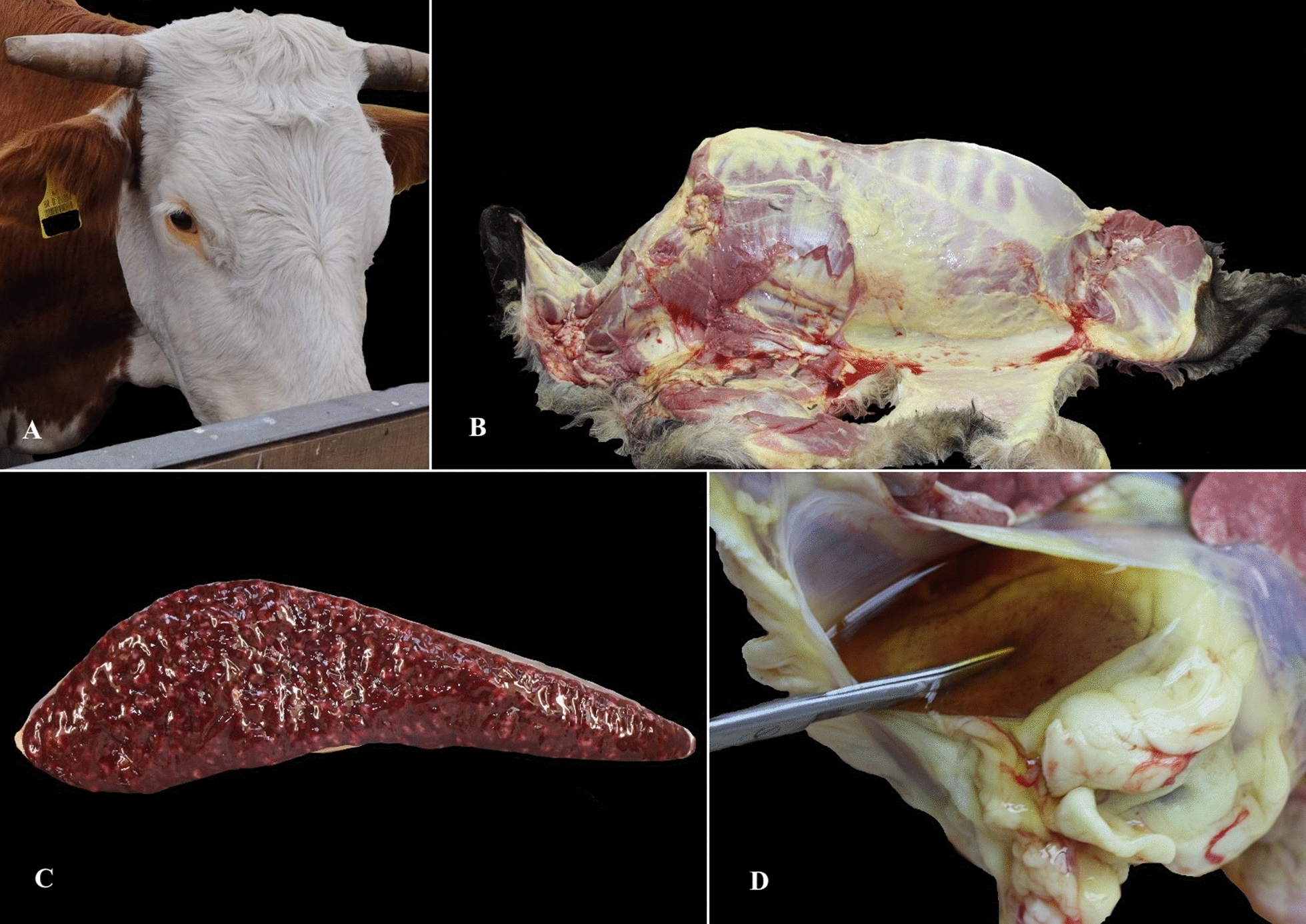
**a** Icterus in a cow infected with *Anaplasma marginale*. **b–d** Post-mortem findings in a sheep infected with *Anaplasma ovis* showing diffuse icterus of subcutaneous fat and fascia (**b**), splenomegaly (**c**) and hydropericardium with epicardial petechiae (**d**)

Duh et al. [70] in 2001 performed a study on piroplasmosis in seven healthy and 10 sick sheep from southern littoral Croatia. Using a molecular approach these authors identified *T. ovis* and *Theileria* sp. OT3 but not *B. ovis* [70]. *Theileria ovis* was present mostly in healthy sheep while *Theileria* sp. OT3 parasite was detected mostly in sick animals; these results were considered evidence of the possible pathogenic nature of *Theileria* sp. OT3. In another study, *A. ovis* was confirmed by the sequencing of msp4 from a sick ram from the Croatian littoral region [81] (Table 3). According to veterinarian practitioners, the described clinical signs of the disease are common in sheep and rams introduced from non-endemic areas and disease has never been observed in animals younger than 5 months. Sheep that died from an *A. ovis* infection frequently exhibited diffuse icterus, splenomegaly, hydropericardium and endocardial petechiae on post-mortem examinations (Fig. 2b–d).

Jurković et al. [82] described two outbreaks of anaplasmosis in Croatian cattle caused by *A. marginale* and by concurrent infection with *A. bovis* and *T. orientalis* complex during June and July. Six *A. marginale* infections in cows from the continental part of Croatia manifested as fever, lethargy, dark urine, ic​ter​us (Fig. 2a) and reddish mucous membranes. Postmortem examination revealed icterus, urinary bladder filled with dark urine and splenomegaly. The sequence of the 840-bp msp4 fragment of the Croatian *A. marginale* isolate clustered with msp4 sequences of *A. marginale* from Russia and Hungary, corresponding to haplogroup 1 detected in Europe, North America, Africa and the Middle East. At almost the same time, *A. bovis* caused a lethal outcome in three cows co-infected with *T. orientalis* (*buffeli/sergenti*) originating from coastal Croatia.

Equine piroplasmosis has been known about for almost a century in Croatia, but* T. equi* (‘*Nuttalia equi*’) was described in 1954 for the first time, recorded in five horses in Northern Croatia in villages close to Zagreb [83]. This finding represents an important discovery since up to this time only *B. caballi* had been detected as a single species in horses from northern Croatia. In their PhD thesis (1954), Richter [84] provided a detailed map of equine piroplasmosis across Croatia. Over a 4-year period, 612 horse blood smears from clinically suspected cases of 'haemosporidia' were microscopically examined. *Babesia caballi* was microscopically confirmed in 403 of these horses, mostly in those from the continental part of the Republic of Croatia, and *T. equi* was found in only one horse blood smear. The author concluded that *B. caballi* is a dominant causative agent of acute clinical piroplasmosis of horses in the territory of the Republic of Croatia. In their PhD thesis (2015), Gotić [85] described 14 acute piroplasmosis cases in horses, of which *B. caballi* was confirmed in 13 horses and *T. equi* in only one case. In the same study on 362 randomly collected, asymptomatic horses, the overall prevalence using a molecular approach and a cellular ELISA (cELISA) was 24.7% [85] (Table 3). A piroplasm DNA was detected in 61/364 horses (16.7%), and further sequencing confirmed the presence of *T. equi* in 48/61 (78.6%) and *B. caballi* in 13/61 (21.3%) samples. Antibodies were present in 92/364 (25.2%) of the horses tested with cELISA. Two genotypes of *T. equi* were detected, genotype E in 10.8% (39/362) of the horses and genotype A in 2.5% (9/362) of the horses, while *B. caballi* was not present. All samples from southern Croatia belonged to the 18S rRNA gene clade A, while samples from northern Croatia were identified as clade *Ehrlichia*. Additionally, the diversity of *T. equi* from Croatia was investigated by analysing the ema-1 gene. All samples from southern Croatia belonging to clade A based on sequencing of the 18S rRNA gene demonstrated *ema-1* homology to the previously described *ema-1* genotype groups A and B. All samples from northern Croatia, previously identified as clade E, assembled distinctly from the previously described *ema-1* groups and clustered into two novel genotype groups, tentatively named *ema-1* groups D and E.

### Wild animals

In a molecular study on spleen samples from 191 carcasses of red foxes, Dežđek et al. [86] discovered four species of haematozoa in 57 foxes (30%) using PCR and sequencing of the 18S RNA gene. *Babesia vulpes* was found in 10 foxes (5.2%), *H. canis* in 44 (23%) foxes, *Hepatozoon* sp. in two foxes (1%) and *T. capreoli* in a single animal (1%) (Table 4). *Babesia vulpes* and *H. canis* were distributed across all the studied regions, while *T. capreoli* and *Hepatozoon* sp. were restricted to the continental area of Zagreb and Zagorje, and Istria regions, respectively. In 2017, Beck et al. [87] performed a pathological and molecular investigation on piroplasm infections in captive and free-ranging grey wolves. PCR amplification targeting the 18S RNA gene revealed the presence of *Theileria/Babesia* DNA in 21 of 108 (19.4%) free-ranging wolves and one captive animal. Subsequent sequencing revealed that 7/108 animals (5.5%) were positive for *B. canis* while 15/22 (13.9%) sequences were found to be identical with those of *T. capreoli* (Table 4). These authors showed that *B. canis* has little impact on wolf health, suggesting that the wolf is the natural host. Hodžić et al. [66] reported a new species of *Hepatozoon* in Croatia, named *H. martis* n. sp., from 64% of samples of European martens (*Martes martes*), as part of a study that included animals from Bosnia and Herzegovina and Croatia. In a large European molecular study on *Hepatozoon* species in wild animal tissue and ticks, *H. canis* was found to be present in 54.2% of gray wolves, 80.8% of golden jackals and 7.8% of badgers in Croatia [68]. The prevalence in small wild mammals varied from 0.6% of *Hepatozoon sciuri* in European hare to 81.8% of *Hepatozoon* sp. in bank voles. *Hepatozoon ayorgbor* was detected in 8.1% of yellow-necked mice and 14.6% of wood mice, and *H. sciuri* was noted in a single European hedgehog (100%) (Table 4).

In their PhD thesis (2012), Pintur [88] analysed spleens from 164 animals, including 49 from roe deer (*Capreolus capreolus*), 102 from red deer (*Cervus elaphus*) and 13 from fallow deer (*Dama dama*), for the presence of piroplasms DNA using conventional PCR that targeted a portion of the 18S rRNA. The overall prevalence was 67.6%, with the highest infection rate found in red deer (56.8%). Across all samples, *Babesia* sp. was detected in 2.9%, *B. capreoli* in 0.9% and *T. capreoli* in 52.9%. *Theileria capreoli* was present in all fallow deer samples, while 22.4% of roe deer samples were positive for *Babesia* (*B. capreoli* 18.3%, *Babesia crassa* 2%, *Babesia venatorum* 2%) and 59.1% were positive for *Theileria* (*T. capreoli* 57.1% and *T. ovis* 2%) (Table 4).

## Montenegro

Data on TBDs in Montenegro are scarce. Although testing has been carried out since the establishment of the health system, there is very little written or published data. Most published data are related to cases of human diseases, while non-zoonotic diseases and their carriers remain unrecorded.

Among the rare studies conducted in Montenegro is a study testing 142 horses in the Central Balkans, which showed a total prevalence of *T. equi* of 22.5% and *B. caballi* of 2.1%. In addition to a few published articles [58], most of the data available today come from unpublished tests by the Diagnostic Veterinary Laboratory and reports from private veterinary clinics (Tables 2, 3). Babesiosis, anaplasmosis and ehrlichiosis are present in livestock (cows, goats and sheep) and pets, especially dogs, as evidenced by annual reports on the work of the Diagnostic Veterinary Laboratory and other rare studies [89–92]. Private veterinary clinics are focused on the treatment of pets and, consequently, data pertaining to the presence of babesia in dogs. In Montenegro, tests on the presence of these diseases in wild animals have never been carried out, so there is no data on their occurrence in wild animals.

## North Macedonia

Non-zoonotic TBPs in North Macedonia have been poorly studied, and there is little published data on this topic. Any available information mainly comes from research carried out in the first half of the twentieth century in domestic animals. In livestock, pathogens such as *B. ovis, T. ovis*, *B. caballi*, *Theileria hirci* and other *Theileria* spp. have been described, often associated with significant impacts on animal health and productivity. The presence of specific pathogens was largely anecdotal, relying on clinical observations of related diseases rather than on confirmed pathogen identification. While the co-occurrence of diseases and competent vectors has been reported, no published studies have confirmed the presence of these pathogens in ticks. No data are available on non-zoonotic TBPs in companion or wild animals, leaving substantial gaps in the epidemiological understanding of these pathogens in North Macedonia.

### Livestock

In a study carried out in 1918, Knuth et al. [93] reported piroplasmosis in German horses that had been returned from the occupied territories in Macedonia in 1917. These authors detected 15 tick species belonging to the family Ixodidae, but only three tick species were identified on the horses suffering from piroplasmosis (*Hyalomma aegyptium, Rh. bursa* and* Rh. sanguineus* s.l.). Concluding that *H. aegyptium* is an improbable vector of piroplasmosis, the authors considered that *Rh. bursa* and *Rh. sanguineus* s.l. are the primary vectors, with particular emphasis on the former. They also hypothesised that *D. reticulatus*, which was predominantly detected on horses in Macedonia in the spring, is a vector of *B. caballi* because of the co-occurrence of babesiosis in horses during the same period of the year.

The first data on piroplasmosis in goats in Macedonia were from Dzunkovski and Urogjevic (cited in Mekuli [94]), who describe the disease agent as *T. hirci*. The authors found Koch plasmatic bodies in the peripheral blood of diseased goats. In 1925, Čolak [17] reported a high prevalence of piroplasmosis among domestic animals in Macedonia. The disease was prevalent in the Kumanovo region, along the Vardar valley, in Ovche Pole, Strumica, Gevgelija, Pelagonia and the surroundings of Ohrid. However, they did not determine the type(s) of etiological agents. In 1933, Marković [95] noted that on Macedonian territory (as part of the Kingdom of Yugoslavia), piroplasmosis occurred in all types of livestock, stating that “*it has not been studied which types of piroplasms are present, but there certainly are many of them*”. According to Mlinac [18], *B. ovis* and *T. ovis* were the causative agents of piroplasmosis in sheep. On the contrary, in a study carried out in 1939, Šterk [22] found that only *T. ovis*, not *B. ovis*, was the causative agent of sheep piroplasmosis. Pavlov [96] reported 16 cases of piroplasmosis in cattle from a village in Macedonia caused by *Theileria* (not specifying the species), and the only tick species found on the infected cattle, but also on the uninfected cattle in the district, was *H. aegyptium*. In two studies [97, 98] carried out in 1955 and 1957, respectively, Angelovski found *B. ovis* to be the causative agent of piroplasmosis in sheep, stating that all diseased sheep were infested with *Rh. bursa*. In a subsequently study, Angelovski [99] confirmed these results during the occurrence of enzootic piroplasmosis in imported sheep and noted that in native sheep, babesiosis occurs sporadically and without clinical signs. In a short review published in 1963, Angelovski et al. [100] presented a brief historical overview of the study of piroplasmosis in Macedonia based on their research on the occurrence, prevalence, clinical signs, gross pathology findings, diagnosis, treatment and prevention. *Babesia ovis* was found to be the causative agent of piroplasmosis in sheep, and *Rh. bursa* was the predominant tick species. In the municipality of Krushevo, Geru [101] also found *B. ovis* in sheep infested with *Rh. bursa,* which presented clinical signs of piroplasmosis. In their first report on babesiosis in the Skopje region in 1987, Geru and Cvetković [102] confirmed the presence of *B. ovis* and *Rh. bursa* in all diseased goats. In their doctoral thesis (1996), Geru [103] tested blood smears from 3800 goats and 1390 juveniles (kids and yearlings) throughout the country and reconfirmed *B. ovis* as the etiological agent of babesiosis in goats (20.7% prevalence in adults; 21.9% in juveniles), with *Rh. bursa* as the main vector (47.8% prevalence in goats)*.* The diseased animals showed mild to severe symptoms, with only 1–3% of parasitised erythrocytes (juveniles had more parasitised erythrocytes than adults). This author experimentally infected goats by through intravenous injections of blood from a diseased sheep. The results showed that the sheep strain of *B. ovis* is also infectious for goats, confirming *B. ovis* as the joint etiological agent of sheep and goat babesiosis.

## Serbia

Most data on non-zoonotic TBPs and TBDs in animals in Serbia focus on babesiosis, which is recognised as the most significant tick-borne animal disease in the country. *Babesia canis*, *B. gibsoni*, *A. ovis*, *A. marginale* and *H. canis* have been identified in ticks, while studies on companion animals have documented a range of pathogens, including *Babesia canis, B. vulpes, B. gibsoni, B. caballi, B. vogeli, A. platys* and *H. canis*. In livestock, pathogens such as *Theileria annulata*, *T. equi, A. marginale,* *B. bigemina* and *B. bovis* are prevalent. Reports from wild animals suggest the presence of *B. canis, B. vulpes* and *H. canis*, though data remain scarce.

### Ticks

In 2003, Pavlović et al. [104] reported the results of a survey conducted between 1997 and 2001, which highlighted the high prevalence of non-zoonotic *B. canis* in various tick species in the Belgrade region. The prevalence proportions of *B. canis* were 66.1% for *Rh. sanguineus* s.l., 46.4% for *D. reticulatus* and 18.7% for *Dermacentor marginatus*. These findings were obtained through microscopic examination of tick smears stained with a 5% Giemsa solution [104]. After utilising microscopic techniques for over a decade to investigate the presence of *Babesia* species in ticks in Serbia, Mihaljica et al. [105] detected, for the first time in 2012, *B. canis* in *D. reticulatus* (21.5%) and *Haemaphysalis concinna* (8.5%) from vegetation by using PCR and sequencing, at the localities of Pančevački Rit, Titov Gaj, Makiš, PKB and Kljajićevo. A similar finding was reported the following year by Tomanović and colleagues [106] (Table 1). Subsequently, Potkonjak et al. [107] detected *B. canis* in 33.3% of examined *D. reticulatus* ticks from dogs in Novi Sad using PCR and sequencing. Meanwhile, Sukara et al. [108] identified *B. canis* DNA in six females of *I. ricinus* collected from golden jackals at three localities (Smederevska Palanka, Surčin, Veliko Gradište) and in one female and seven males of *D. reticulatus* from three localities (Smederevo, Surčin, Titel) (Table 1). In addition to the detection of *B. canis* in ticks within Serbia, it is noteworthy that Davitkov et al. [109] reported the first identification of *B. gibsoni* in two *Rh. sanguineus* s.l. ticks (4.1%) using the PCR–restriction fragment length polymorphis (RFLP) method in 2016 (Table 1). These authors also noted that 14.2% of the samples lacked a restriction site for any of the enzymes used, effectively ruling out the presence of species such as *Babesia rossi*, *B. vogeli*, and *B. microti*-like and thus indicating a high likelihood of *B. canis* presence. Also, they reported the detection of *Babesia* spp. in ticks collected from asymptomatic dogs in three Belgrade municipalities (Savski venac, Novi Beograd and Zemun), with an overall prevalence rate of 18.3%. The prevalence proportions of *Babesia* spp. were 44.4% for *D. reticulatus*, 12.9% for *Rh. sanguineus* s.l. and 11.1% for *I. ricinus* [109].

*Anaplasma ovis* was found in questing ticks collected by Sukara et al. from localities in the northern part of Serbia in the period 2007–2009 [108]. These authors used the PCR method and subsequent sequencing (Table 1), reporting that the prevalence of *A. ovis* in *Hae. concinna* ticks was 20%, increasing up to 50% in *Hae. punctata* ticks, and that in *I. ricinus*, this pathogen was present in 29.6% of analysed ticks. .

*Hepatozoon canis* has been confirmed in *I. ricinus* ticks collected from dogs [107]. Since *I. ricinus* is not considered to be a competent vector for *H. canis*, the authors suggested that the tick became infected through a blood meal. The presence of *H. canis* DNA was also detected in *Rh. sanguineus* s.l., a tick species recognised as a competent vector, when a positive tick was removed from a dog [110]. Non-zoonotic pathogens from the genus *Anaplasma* have been described several times in Serbia in the last decade. *Anaplasma marginale* was identified by Sukara and colleagues in 6.4% *D. reticulatus* ticks collected from golden jackals in Surčin and Smederevo localities between 2010 and 2013 [108] (Table 1).

### Companion animals

Although the clinical description of babesiosis in animals in Serbia has been known since the nineteenth century, and the first microscopic identification of piroplasm in dogs' blood dates back to 1953, awareness of the importance of babesiosis in dogs occurred only in the 1980s with the development of more intensive diagnostic methods as well as follow-up programmes and investigations of *Babesia* species [111–113]. Thirty years ago, babesiosis was recognised as a prevalent canine disease in Serbia [113]. Since then, extensive research has been conducted on dog populations to detect and characterise *Babesia* species, as evidenced by the significant number of publications. In a comprehensive 5-year-long study (1997–2001) involving 3945 pet dogs with clinical symptoms (anaemia, haemoghlobinuris, fever, paleness) or tick infestation, all from the Belgrade area, Pavlović et al. [104] found that the prevalence of *B. canis* was 74.1% using microscopic examination of stained blood smears. In a subsequent study and applying methodology similar to that of their previous research, Pavlović et al. [114] observed a significantly lower prevalence of 34.9%. Savić et al. [115] reported the presence of *Babesia* species in 11.7% of dogs in the Vojvodina region (northern Serbia) in 2012, with an increase to 12.5% in 2013, using microscopic examination of stained blood smears. Davitkov et al. [116] conducted a study (period 2012–2014) in Serbia using sequencing and confirmed the presence of *B. canis* and *B. gibsoni* in symptomatic dogs exhibiting clinical findings of babesiosis for the first time (Table 2). These authors considered both cases to be autochthonous infections because the dogs were born in Serbia and had never been taken abroad. In a study conducted in 2018, Kovačević-Filipović et al. [117] found that 13.5% of clinically healthy dogs residing in suburban and rural areas of Belgrade municipalities tested positive for *B. canis* using PCR, with 2.7% of dogs positive for *B. gibsoni*. Interestingly, in their cross-sectional survey on dogs, which was carried out during the period 2012–2014, Gabrielli et al. [118] reported *B. vulpes* (10.1% of dogs), *B. gibsoni* (4.5%), *B. vogeli* (1.9%) and *B. caballi* (1.9%) but not *B. canis*. Regarding the spatial distribution, *B. vulpes* was exclusively found in Prokuplje, located in the southern region of Serbia, and *B. vogeli* and *B. caballi* were exclusively detected in Pančevo, which is near Belgrade. *Babesia gibsoni* was identified in both of these cities [118]. Only a few seroepidemiological studies on babesiosis in dogs have been carried out in Serbia. Potkonjak et al. [119] found 26.1% *B. canis* seropositive dogs in Novi Sad, Vojvodina region (northern Serbia) using IFAT. Also using IFAT, in the same region in 2015, Spasojević-Kosić et al. [120] reported a slightly higher seroprevalence of 32.7% based on their detection of seropositive hunting dogs with the same methodology. In 2018, in the Belgrade region, Kovačević-Filipović et al. [117] reported different findings, noting that 51.4% of dogs were seroreactive to *B. canis*, 12.6% to *B. gibsoni* and 52.3% to *B. vogeli* using the IFAT. Janjić et al. [120] demonstrated a bimodal seasonal distribution of canine babesiosis, characterised by a significant peak in the spring and a less prominent one in the autumn. In 2020, Potkonjak et al. [122] reported that *B. canis* is endemic in Serbia, with frequent local transmission and a high expected frequency of clinical disease in dogs, and they also mentioned that *B. gibsoni* and *B. vogeli* have rare local transmission, affecting risk areas with an intermediate expected frequency of clinical disease in dogs.

To date, no data on the presence of *A. platys* in animals are available, but this pathogen has been detected in dogs from Serbia through molecular and serological analysis. The study by Ilić Božović and colleagues [123] from 2018 reports the molecular detection of the pathogen in one dog, while specific antibodies against *A. platys* were also detected by the SNAP assay (SNAP® M-A; IDEXX Laboratories, Inc., Westbrook, MA, USA) in one dog out of 111 animals, with a prevalence of 0.9% [117].

The clinical significance of *H. canis* infection in dogs was demonstrated in 2023 by Sukara and colleagues [124] when these researchers confirmed hepatozoonosis in an 8-year-old Miniature Schnauzer, while a 4-year-old mixed breed male dog with clinical symptoms was proven to be co-infected with *H. canis* and *E. canis*.

### Livestock

Over the years, research findings have highlighted the significant role of non-zoonotic *Babesia* species in veterinary medicine. In their molecular survey, published in 2016, Davitkov et al. [58] identified a prevalence of 1.1% for *B. caballi* in 94 apparently healthy horses. In 2022, Pavlović et al. [125] claim to have detected *B. bigemina* and *B. bovis* in 3.6% and 5.7%, respectively, of the tested cattle using microscopic examination of stained blood smears. As these findings were not confirmed by molecular tests, they should be considered with caution (Table 3). The first report of theileriosis in this region dates back to 1924, when *T. hirci* (*lestoquardi*) was detected in goats, sheep and cattle in the area of present-day North Macedonia, which at that time belonged to the Kingdom of Serbs, Croats and Slovenes [126]. Almost 100 years later, the second case of theileriosis in cattle was described by Pavlović and Dimitrijević in 2020 [127] on the territory of present-day Serbia. This pathogen was identified as *T. annulata* using stained blood smears. In another study, *Theileria annulata* was detected in 1.4% of blood samples taken from cattle with clinical signs of theileriosis using light microscopy examination of stained blood smears. A total of 572 animals from 61 villages (Kolubara, Mačva, Braničevo, Podunavlje and Zaječar districts in Serbia) were included in the study [125]. Molecular epizootiology studies on *Theileria* spp. are relatively recent. In blood samples from horses and donkeys, *T. equi* was identified in 26/84 horses, while *Theileria caballi* was not confirmed in any of the tested horses from the Serbian region. The prevalence of *T. equi* detected in horses was 27.7% [58] (Table 3). In a follow-up investigation involving 70 apparently healthy donkeys (from the localities of Zasavica, Stara Planina and Kovilj), Davitkov and colleagues [128] documented a total prevalence of *T. equi* infection of 50% using PCR and sequencing. In the 2018 study of Vasić et al. [129], *Theileria* spp. was detected in cattle with a prevalence of 3.7%. PCR products were sequenced and identified with 100% identity with GenBank entries from Italy (*T. sergenti*), China (*Theileria* spp.) and Korea (*Theileria buffeli* isolate HS252). In 2022, Pavlović and colleagues [125] reported cases of anaplasmosis in 11.9% of milk cattle in the Beljanica mountains.

### Wild animals

Two papers have been published reporting the presence of *Babesia* species in wild animals. Sukara et al. [108], in a recent study investigating the reservoir potential of golden jackals and their roles in enzootic cycles in Serbia, detected *B. canis* DNA in 4.2% of spleen samples (Table 4). Juwaid et al. [130] documented the presence of *B. vulpes* (28.7%) and *B. canis* (0.8%) in red foxes using molecular biological methods.

In 2014, Duscher et al. [131] reported for the first time *H. canis* in Serbia in samples from golden jackals (*Canis aureus*). The liver or skeletal muscle tissue of 206 golden jackals was screened by PCR, and 67.5% of analysed animals tested positive. The presence of *H. canis* was subsequently confirmed in the blood of a clinically healthy dog in Niš (southern Serbia) [118]. More recent studies on wild canids revealed a high prevalence of the pathogen in analysed spleen samples from red foxes (*Vulpes vulpes*) and grey wolves (*Canis lupus*). A total of 61.2% of analysed foxes [130] and 57.9% of analysed wolves [132] tested positive. The results of a recently published study (2024) have also identified the presence of *H. canis* in small rodents. Using PCR followed by sequencing, the DNA of *H. canis* has been detected in one *Apodemus flavicolis* (0.9%) mice representing first finding of *H. canis* in small rodents worldwide [133].

## Conclusions

This study encompasses nearly a century, from initial investigations with early descriptions of piroplasms and anaplasmas to current research mostly concentrating on the molecular identification of TBDs. Data on TBDs in the WBs are intrinsically linked to historical events resulting from border alterations following the First and Second World Wars, as well as the establishment of new states, such as the Kingdom of Yugoslavia, followed by the Federative People's Republic, the Socialist Republic of Yugoslavia and ultimately the disintegration of Yugoslavia.

Given the significance of TBDs in ruminants, systematic data collection was established with the primary objective of mitigating substantial economic losses and enhancing animal output more than a century ago. Surveillance of pathogens and descriptions of *Babesia, Theileria* and *Anaplasma* served as foundational elements for the implementation of control strategies. One of the most important of these early findings was that indigenous cattle and sheep exhibited a greater resistance due to their adaptation to piroplasms, while imported animals developed significant clinical symptoms, followed by succumbing to piroplasmosis. The early descriptions of *Babesia* and *Theileria* species in cattle regarding morphological specificity were likely incorrect as currently only three species—*T. orientalis*, *Anaplasma bovis* and *A. marginale*—have been documented in cattle in the WBs. The descriptions of *T. mutans*, *T. dispar* or *T. parva* possibly correspond to the already recognised *T. orientalis* in cattle from Croatia, Serbia and Bosnia and Herzegovina. The presence of species such as *Babesiella bovis*, *B. maior* n.sp., *B. berbera* and *P. bigeminum* remains uncertain.

Comprehensive molecular investigations across the WBs are essential to explore the potential variety of TBDs, particularly in southern Serbia and North Macedonia, as also observed a century ago. Overall, studies on TBDs in cattle in the WBs are inadequate and fail to provide an accurate assessment of their prevalence or significance. The circumstances closely resemble current understanding of TBDs in small ruminants. Aside from the molecularly confirmed examples of *A. ovis* in Croatia and *B. ovis* in Bosnia and Herzegovina in sheep, systematic research is lacking, despite the strong likelihood that the causal agents are present throughout the WBs. It is noteworthy that there is a complete absence of data on pathogens in goats.

The old descriptions of piroplasms in horses are accurate; however, the official terminology has been updated (*P. caballi* is now *B. caballi*, and *N. equi* is now *T. equi*). A few recent studies have confirmed the coexistence of both *T. equi* and *B. caballi*, along with the identification of two entirely novel genotypes of *T. equi* in Croatia. Despite the disease being documented and objectively verified a century ago, there remains a shortage of molecular research to ascertain genetic variation in this region.

Unlike studies on ruminants and horses, there is a significantly larger body of research on dogs, which clearly highlights the evolving relevance and role of pets in comparison to livestock. Studies on canine piroplasm species revealed the first identification of *B. vulpes* (*T. annae*) outside of Spain. Most investigations have identified *B. canis* as the predominant pathogenic species due to its pathogenicity and distribution. The number of molecular studies has resulted in the detection of dog-specific *B. canis, B. vogeli, B. gibsoni* and * B. vulpes*, but also of non-host-specific *T. equi* and *B. caballi*. It would therefore appear that current knowledge of dog piroplasm species is sufficient. Comprehensive research on dogs has facilitated the identification of *H. canis*, *E. canis* and *A. platys*; however, the distribution and prevalence in certain regions require further investigation. All dog TBDs identified in other parts of Europe have been verified in the WBs.

With regard to wild animals, it is noteworthy that the majority of research has focused on wild canines and carnivores, with only one study having documented non-zoonotic tick-borne microorganisms in artiodactyl species and wild felids. The likely reason for this lack of data is that scientific interest has predominantly focused on detecting animal reservoirs of zoonotic tick-borne microorganisms, rather than on non-zoonotic ones. Descriptions of *H. silvestris*, *H. martis* and *C. europaeus* have demonstrated the diversity of tick-transmitted species. Moreover, studies detecting *B. canis, B. vulpes* and *H. canis* have suggested the significance of wild canines as reservoirs for domestic animals.

With the exception of a few studies detailing *B. canis, A. ovis* and *H. canis* in questing ticks, the majority of research has focused on TBPs in ticks collected from hosts.

*Babesia divergens* and *Anaplasma phagocytophilum* were excluded from this review due to their established zoonotic potential, while *E. canis* was included because its ability to infect humans is not well-understood. Recent evidence suggests that certain *E. canis* genotypes may have zoonotic potential, with cases predominantly reported in the Americas [134, 135]. Therefore, the inclusion of *E. canis* in the review reflects the need for further investigation into its potential risk to human health, especially in areas where *R. sanguineus* is widespread.

Comprehensive research is essential in the WBs due to the extensive historical migrations of both humans and animals, coupled with a lack of current studies. It is crucial to highlight that all WB countries have established national animal disease monitoring programmes, as mandated by the EU. However, there are no national monitoring systems for TBDs, despite their significance, particularly in the context of climate change and alterations in outdoor animal husbandry and traditional small ruminant breeding. In contrast to the previous comprehensive monitoring systems for TBDs in ruminants and horses, these diseases are currently unreported, with the categorisation of neglected diseases, a situation we consider to be fully unjustified. Research on zoonotic infections and those 'potentially' zoonotic appears to be more attractive; nonetheless, we believe that non-zoonotic VBDs in the WBs warrant more attention due to their significance and potential impact on biodiversity.

## Data Availability

No datasets were generated or analysed during the current study.

## Abbreviations

ELISA: Enzyme-linked immunosorbent assay
FFPEB: Paraffin-embedded tissue blocks
IFAT: Indirect immunofluorescence test
PCR-RFLP: Restriction fragment length polymorphism-PCR
qPCR: Real-time PCR
RLBH: Reverse line blot hybridisation
TBD: Tick-borne disease
TBP: Tick-borne pathogen
VBD: Vector-borne disease
VBP: Vector-borne pathogen
WB: Western Balkans

## References

[1] Dantas-Torres F, Chomel BB, Otranto D. Ticks and tick-borne diseases: a one Health perspective. Trends Parasitol. 2012;28:437–46. 22902521 10.1016/j.pt.2012.07.003

[2] Kernif T, Leulmi H, Raoult D, Parola P. Emerging tick-borne bacterial pathogens. Microbiol Spectr. 2016;4:295–310. 10.1128/microbiolspec.EI10-0012-2016 27337487

[3] Otranto D, Dantas-Torres F. Canine and feline vector-borne diseases in Italy: current situation and perspectives. Parasit Vectors. 2010;3:1–12. 20145730 10.1186/1756-3305-3-2 PMC2818618

[4] Baneth G. Tick-borne infections of animals and humans: a common ground. Int J Parasitol. 2014;44:591–6. 24846527 10.1016/j.ijpara.2014.03.011

[5] Gilbert L. The impacts of climate change on ticks and tick-borne disease risk. Annu Rev Entomol. 2021;66:373–88. 33417823 10.1146/annurev-ento-052720-094533

[6] Eskezia BG, Desta AH. Review on the impact of ticks on livestock health and productivity. J Biol Agric Healthcare. 2016;22:2224–3208.

[7] Hofmeester TR, Sprong H, Jansen PA, Prins HHT, van Wieren SE. Deer presence rather than abundance determines the population density of the sheep tick, *Ixodes**ricinus*, in Dutch forests. Parasit Vectors. 2017;10:433. 28927432 10.1186/s13071-017-2370-7 PMC5606071

[8] Kazimírová M, Hamšíková Z, Špitalská E, Minichová L, Mahríková L, Caban R, et al. Diverse tick-borne microorganisms identified in free-living ungulates in Slovakia. Parasit Vectors. 2018;11:1–18. 30176908 10.1186/s13071-018-3068-1 PMC6122462

[9] Lindsø LK, Dupont P, Rød-Eriksen L, Andersskog IPØ, Ulvund KR, Flagstad Ø, et al. Estimating red fox density using non-invasive genetic sampling and spatial capture–recapture modelling. Oecologia. 2022;198:139–51. 34859281 10.1007/s00442-021-05087-3 PMC8803778

[10] Massei G, Kindberg J, Licoppe A, Gačić D, Šprem N, Kamler J, et al. Wild boar populations up, numbers of hunters down? A review of trends and implications for Europe. Pest Manag Sci. 2015;71:492–500. 25512181 10.1002/ps.3965

[11] Martin JL, Chamaillé-Jammes S, Waller DM. Deer, wolves, and people: costs, benefits and challenges of living together. Biol Rev. 2020;95:782–801. 32043747 10.1111/brv.12587

[12] Gortázar C, Acevedo P, Ruiz-Fons F, Vicente J. Disease risks and overabundance of game species. Eur J Wild Res. 2006;52:81–7.

[13] Mikačić D. Ticks in the littoral belt of Yugoslavia III. Distribution and dynamics of species in the course of the year. Vet Arh. 1965;35:155–70.

[14] La IU. Piroplasmosi in Dalmazia (Forme, profilassi e cura). Clin Vet. 1921;44:447–54.

[15] Petrović DM. Proučavanje piroplazmoze kod nas. Jugosl vet Glasn. 1922;7:14–5.

[16] Džunkovski E. O radovima Antimalarične Komisije Ministarstva Narodnog Zdravlja, koji se tiču svakovrsnih protozojskih bolesti domaće stoke i ptica. Jugosl vet Glasn. 1923;4:55–6.

[17] Čolak M. Nekoliko riječi o piroplazmozi u Južnoj Srbiji. Jugosl vet Glasn. 1925;12:166–7.

[18] Mlinac F. Deset godina veterinarskog laboratorijskog, epizootiološkog i higijenskog rada u Južnoj Srbiji. Jugosl vet Glasn. 1937;10:399–408.

[19] Mlinac F, Petrović DM, Babuder C. Prilog proučavanju piroplasmoza u Vardarskoj banovini Piroplazme Goveda. Jugosl Vet Glasn. 1934;8:389–420.

[20] Mlinac F, Šterk V. Anaplazmoza goveda u Južnoj Srbiji. Jugosl Vet Glasn. 1937;1:1–9.

[21] Petrović DM. Piroplasmoze konja. Jugosl Vet Glasn. 1935;7:349–68.

[22] Šterk V. Izveštaj o radu veterinarskog odelenja higijenskog zavoda u Skoplju za 1938 godinu. Jugosl vet Glasn. 1939;6:259–64.

[23] Horvatić I. Izvještaj o radu veterinarskog odelenja pri Higijenskom zavodu u Skoplju za 1939 god. Jugosl vet Glasn. 1940;5:159–64.

[24] Ranitović M. Piroplazmoza ovaca u Banatu (*Piroplasmosis**ovium*). Jugosl vet Glasn. 1929;10:321–3.

[25] Ćosić F. Piroplasmosa goveda u srezu ključkom Liječenje acaprinom u 1937/38 god. Jugosl vet Glasn. 4 187–188.

[26] Babić I. Izvještaj Zavoda za patološku anatomiju veterinarskog fakulteta u Zagrebu za god. Jugosl vet Glasn. 1931;7:196–8.

[27] Winterhalter M. Razudbeni nalazi kod uginulih konja razuđenih u toku godina 1928–1938 u Zavodu za patološku anatomiju Veterinarskog fakulteta u Zagrebu. Jugosl vet Glasn. 1939;4:169–74.

[28] Winterhalter M. Razudbeni nalazi kod uginulih goveda razuđenih u toku godina 1928–1938 u Zavodu za patološku anatomiju Veterinarskog fakulteta u Zagrebu. Jugosl vet Glasn. 1939;8:357–60.

[29] Plasaj S. Izveštaj o poslovanju zavoda za nauku o zarazama vet fakulteta u Zagrebu u školskoj godini 1927–28. Jugosl vet Glasn. 1929;3:69–72.

[30] Plasaj S. Izveštaj o poslovanju zavoda za nauku o zarazama vet fakulteta u Zagrebu u školskoj godini 1928–29. Jugosl vet Glasn. 1930;2:31–4.

[31] Rajčević MI, Butozan V. Prilog liječenju goveđe piroplazmoze todoritom. Jugosl vet Glasn. 1932;10:373–82.

[32] Sutlić A. Piroplazmoza (babezioza) pasa. Vet Arh. 1942;7:308–13.

[33] Boko F. Piroplazmoza u srednjoj Dalmaciji. Jugosl vet Glasn. 1941;2:65–8.

[34] Christova I, Van De Pol J, Yazar S, Velo E, Schouls L. Identification of *Borrelia**burgdorferi* sensu lato, *Anaplasma* and *Ehrlichia* species, and spotted fever group rickettsiae in ticks from Southeastern Europe. Eur J Clin Microbiol Infect Dis. 2003;22:535–42. 12938010 10.1007/s10096-003-0988-1

[35] Silaghi C, Knaus M, Hamel D, Rapti D, Pfister K, Rehbein S. Molecular detection of pathogens in ticks and fleas infesting dogs in Albania. In: Abstracts 12th International Symposium on Ectoparasites in Pets, 7-10 April 2013, Munich; p 35.

[36] Koleci X, Keçi R, Zalla P, Farkas R. Piroplasms detected by PCR in ticks collected from goats in Albania. Conference paper. In: Final Capara Conference & MC meeting, 2-4 December 2013, Berlin.

[37] Dhamo G, Rapti D, Bizhga B, Llazari A. Kërkime hematologjike paraprake mbi babezionën e qene preliminary hematologic research on canine babesiosis. Revista Shqiptare e Shkencave Bujqësore. 2006;5:114–9.

[38] Bizhga B, Rapti D, Bejleri B, Xhemali E. Kërkime rreth ehrlichozës ne qen në rretin e Tiranës. Study on canine ehrlichiosis in the Tirana area. Poster at Symposium ‘Kerkimi shencor dhe aplikimet ne Veterinari’, 26 Oct 2006, Tirana.

[39] Lazri T, Duscher G, Edelhofer R, Bytyci B, Gjino P, Joachim A. Infektion mit arthropodenübertragenen Parasiten bei Hunden im Kosovo und in Albanien unter besonderer Berücksichtigung der Leishmanieninfektionen. Wien Klin Wochenschr. 2008;120:54–8. 10.1007/s00508-008-1076-4 19066774

[40] Vercammen F, De Deken R, Maes L. Clinical and serological observations on experimental infections with *Babesia**canis* and its diagnosis using the IFAT. Parasite. 1995;2:407410. 8745740

[41] Hamel D, Silaghi C, Knaus M, Visser M, Kusi I, Rapti D, et al. Detection of *Babesia**canis* subspecies and other arthropod-borne diseases in dogs from Tirana Albania. Wien Klin Wochenschr. 2009;121:42–5. 19915816 10.1007/s00508-009-1234-3

[42] Hamel D, Shukullari E, Rapti D, Silaghi C, Pfister K, Rehbein S. Parasites and vector-borne pathogens in client-owned dogs in Albania Blood pathogens and seroprevalences of parasitic and other infectious agents. Parasitol Res. 2016;115:489–99. 26453093 10.1007/s00436-015-4765-8

[43] Shukullari E, Rapti D, Visser M, Pfister K, Rehbein S. Parasites and vector-borne diseases in client-owned dogs in Albania: infestation with arthropod ectoparasites. Parasitol Res. 2017;116:399–407. 27796564 10.1007/s00436-016-5302-0

[44] Andoni E, Rapti D, Postoli R, Zalla P. Hematologic changes in dogs naturally infected with *Babesia*. AJAS. 2012;11:155–8.

[45] Andoni E, Rapti D, Postoli R, Dimco E, Abeshi J. Clinicopathological findings in naturally infected dogs with *Babesia*. AJAS. 2013;12:185–9.

[46] Petrovec M, Sixl W, Marth E, Bushati N, Wüst G. Domestic animals as indicators of *Anaplasma* species infections in Northern Albania. Ann N Y Acad Sci. 2003;990:112–5. 12860610 10.1111/j.1749-6632.2003.tb07347.x

[47] Zalla P, Shoshi N, Dini V, Bizhga B. Prevalence of cattle babesiosis and anaplasmosis infection in Kruja. Aktet e Takimit Vjetor. Instituti Alb-Shkenca 2008; Vëll. II(3).

[48] Goletić T, Klarić Soldo D, Kapo N, Goletić Š, Koro-Spahić A, Alispahić A, et al. Tick-Borne Pathogens in *Dermacentor**reticulatus* Ticks from Bosnia and Herzegovina. Pathogens. 2024;13:421. 38787273 10.3390/pathogens13050421 PMC11123776

[49] Omeragić J, Kapo N, Goletić Š, Softić A, Terzić I, Šabić E, et al. Investigation of Tick-Borne Pathogens in *Ixodes* Ticks from Bosnia and Herzegovina. Animals. 2024;14:2190. 39123716 10.3390/ani14152190 PMC11311058

[50] Omeragić J, Zuko A, Jažić A, Šaljić E, Škapur V, Habota A, Tick borne babesiosis of dogs in Bosnia and Herzegovina. In: Proceedings 27th World Veterinary Congress 2002, 25–29 September 2002, Tunis.

[51] Omeragić J, Hrvat H. Occurrence of protozoa in dogs in the area of Tuzla. In: International Congress on One World-One Health-One Vision, 14-16 October 2015, Sarajevo. p. 14–5.

[52] Majkić M, Kovačević V, Kovčević D. Prevalenca babezioze pasa na teritoriji općine Teslić (in Bosnian). Vet J Rebublic Srpska. 2015;2:191–299.

[53] Ćoralić A, Gabrielli S, Zahirović A, Stojanović NM, Milardi GL, Jažić A, et al. First molecular detection of *Babesia**canis* in dogs from Bosnia and Herzegovina. Ticks Tick-borne Dis. 2018;9:363–8. 29290581 10.1016/j.ttbdis.2017.11.013

[54] Maksimović Z, Dervišević M, Zahirović A, Rifatbegović M. Seroprevalence of *Anaplasma* spp. and *Ehrlichia* spp. and molecular detection of* Anaplasma phagocytophilum* and* Anaplasma platys* in stray dogs in Bosnia and Herzegovina. Ticks Tick Borne Dis. 2022;13:101875. 34894522 10.1016/j.ttbdis.2021.101875

[55] Colella V, Huggins L, Hodžić A, Galon C, Traub R, Alić A, et al. High-throughput microfluidic real-time PCR for the simultaneous detection of selected vector-borne pathogens in dogs in Bosnia and Herzegovina. Transbound Emerg Dis. 2022;69:e2943–51. 35766324 10.1111/tbed.14645 PMC9796230

[56] Kozinc M. Prinosi ka početnom istraživanju “šimitre” u Hercegovini zvanom ‘leđanica.’ Jugoslavenski veterinarski glasnik. 1936;5:252 (in Bosnian)

[57] Papić Lj: Prilog poznavanju piroplazmoze goveda i njenih vektora na području općine Bugojno. Sarajevo: Magistarski rad; 1976.

[58] Davitkov D, Vucicevic M, Stevanović J, Krstić V, Slijepčević D, Glavinić U, et al. Molecular detection and prevalence of *Theileria**equi* and *Babesia**caballi* in horses of central Balkan. Acta Parasitol. 2016;61:337–42. 27078657 10.1515/ap-2016-0044

[59] Stevanović O, Radalj A, Subić I, Jovanović NM, Sladojević Ž, Amović M, et al. The presence of malignant ovine babesiosis in Bosnia and Herzegovina indicates a possible emerging risk for Balkan region. Comp Immunol Microbiol Infect Dis. 2022;90:101893. 36240662 10.1016/j.cimid.2022.101893

[60] Stevanović O, Radalj A. Molecular evidence of* Theileria orientalis* infection in cattle from Bosnia and Herzegovina. Vet Glas. 2023;77:80–6.

[61] Stevanović O, Ilić T, Jovanović N, Vejnović B, Radalj A. High genetic diversity of Anaplasma ovis in sheep from Bosnia and Herzegovina. Mol Biol Rep. 2024;51:936. 39182201 10.1007/s11033-024-09869-9

[62] Hodžić A, Alić A, Fuehrer HP, Harl J, Wille-Piazzai W, Duscher GG. A molecular survey of vector-borne pathogens in red foxes (*Vulpes**vulpes*) from Bosnia and Herzegovina. Parasit Vectors. 2015;8:1–7. 25889961 10.1186/s13071-015-0692-x PMC4367825

[63] Hodžić A, Alić A, Prašović S, Otranto D, Baneth G, Duscher GG. *Hepatozoon**silvestris* sp. nov.: morphological and molecular characterization of a new species of* Hepatozoon* (Adeleorina: Hepatozoidae) from the European wild cat (*Felis silvestris silvestris*). Parasitology. 2017;144:650–61. 27938443 10.1017/S0031182016002316 PMC5426326

[64] Hodžić A, Alić A, Duscher GG. High diversity of blood-associated parasites and bacteria in European wild cats in Bosnia and Herzegovina: a molecular study. Ticks Tick Borne Dis. 2018;9:589–93. 29422447 10.1016/j.ttbdis.2018.01.017

[65] Panait LC, Mihalca AD, Modrý D, Juránková J, Ionică AM, Deak G, et al. Three new species of* Cytauxzoon* in European wild felids. Vet Parasitol. 2021;290:109344. 10.1016/j.vetpar.2021.109344 33465567

[66] Hodžić A, Alić A, Beck R, Beck A, Huber D, Otranto D, et al. *Hepatozoon**martis* n. sp. (Adeleorina: Hepatozoidae): Morphological and pathological features of a* Hepatozoon* species infecting martens (family Mustelidae). Ticks Tick Borne Dis. 2018;9:912–20. 29605549 10.1016/j.ttbdis.2018.03.023

[67] Alić A, Šupić J, Goletić T, Rešidbegović E, Lutvikadić I, Hodžić A. A unique case of fatal coinfection caused by *Leptospira* spp. and *Hepatozoon**canis* in a Red Fox Cub (*Vulpes vulpes*). Pathogens. 2021;11:11. 35055959 10.3390/pathogens11010011 PMC8777892

[68] Uiterwijk M, Vojta L, Šprem N, et al. Diversity of *Hepatozoon* species in wild mammals and ticks in Europe. Parasit Vectors. 2023;16:27. 36694253 10.1186/s13071-022-05626-8 PMC9872412

[69] Beck R, Habrun B, Bosnić S, Benić M, Nemeth-Blažić T, Barišin S. Identification of pathogens in *Ixodes ricinus* and *Dermacentor reticulatus* from public gardens in Zagreb, Croatia. In Proceedings of the 12th International Conference on Lyme Borreliosis and Other Tick-Borne Diseases, 6–29 November 2010, Ljubljana. p. 95.

[70] Duh D, Punda-Polic V, Trilar T. Molecular detection of *Theileria* sp. in ticks and naturally infected sheep. Vet Parasitol. 2008;151:327–31. 18158215 10.1016/j.vetpar.2007.11.004

[71] Cacciò SM, Antunović B, Moretti A, Mangili V, Marinculić A, Barić RR, et al. Molecular characterisation of *Babesia**canis**canis* and *Babesia**canis**vogeli* from naturally infected European dogs. Vet Parasitol. 2002;106:285–92. 12079734 10.1016/s0304-4017(02)00112-7

[72] Beck R, Vojta L, Mrljak V, Marinculić A, Beck A, Živičnjak T, et al. Diversity of *Babesia* and *Theileria* species in symptomatic and asymptomatic dogs in Croatia. Int J Parasit. 2009;39:843–8. 10.1016/j.ijpara.2008.12.005 19367832

[73] Brkljačić M, Matijatko V, Kiš I, Kučer N, Foršek J, Rafaj Barić R, et al. Molecular evidence of natural infection with *Babesia**canis**canis* in Croatia. Acta Vet Hung. 2010;58:39–46. 20159737 10.1556/AVet.58.2010.1.4

[74] Beck A, Huber D, Antolić M, Anzulović Ž, Reil I, Polkinghorne A, et al. Retrospective study of canine infectious haemolytic anaemia cases reveals the importance of molecular investigation in accurate postmortal diagnostic protocols. Comp Immunol Microbiol Infect Dis. 2019;65:81–7. 31300132 10.1016/j.cimid.2019.05.006

[75] Huber D, Beck A, Anzulović Ž, Jurković D, Polkinghorne A, Baneth G, et al. Microscopic and molecular analysis of *Babesia**canis* in archived and diagnostic specimens reveal the impact of anti-parasitic treatment and postmortem changes on pathogen detection. Parasit Vectors. 2017;10:1–9. 29047398 10.1186/s13071-017-2412-1 PMC5648450

[76] Vojta L, Mrljak V, Curković S, Zivicnjak T, Marinculić A, Beck R. Molecular epizootiology of canine hepatozoonosis in Croatia. Int J Parasitol. 2009;39:1129–36. 19249302 10.1016/j.ijpara.2009.02.007

[77] Dyachenko V, Pantchev N, Balzer HJ, Meyersen A, Straubinger RK. First case of *Anaplasma**platys* infection in a dog from Croatia. Parasit Vectors. 2012;5:1–7. 22401583 10.1186/1756-3305-5-49 PMC3315729

[78] Mrljak V, Kuleš J, Mihaljević Ž, Torti M, Gotić J, Crnogaj M, et al. Prevalence and geographic distribution of vector-borne pathogens in apparently healthy dogs in Croatia. Vector Borne Zoonotic Dis. 2017;17:398–408. 28448211 10.1089/vbz.2016.1990

[79] Jurković D, Beck A, Huber D, Mihaljević Ž, Polkinghorne A, Martinković F, et al. Seroprevalence of vector-borne pathogens in dogs from Croatia. Parasitol Res. 2019;118:347–52. 30377795 10.1007/s00436-018-6129-7

[80] Huber D, Reil I, Duvnjak S, Jurković D, Lukačević D, Pilat M, et al. Molecular detection of *Anaplasma**platys*, *Anaplasma**phagocytophilum* and *Wolbachia* sp. but not* Ehrlichia canis* in Croatian dogs. Parasitol Res. 2017;116:3019–26. 28905230 10.1007/s00436-017-5611-y

[81] Šarić T, Beck A, Taraš I, Šuto A, Orlović D, Jurković D, et al. Prvi dokaz rikecije *Anaplasma ovis* u oboljelog ovna:od kliničkih znakova bolesti do genske tipizacije uzročnika. Vet Stanica. 2022;53:549–560. 10.46419/vs.53.5.9.

[82] Jurković D, Mihaljević Ž, Duvnjak S, Silaghi C, Beck R. First reports of indigenous lethal infection with *Anaplasma**marginale*, *Anaplasma**bovis* and *Theileria**orientalis* in Croatian cattle. Ticks Tick Borne Dis. 2020;11:101469. 32723641 10.1016/j.ttbdis.2020.101469

[83] Petrović D, Kralj M. Nutalioza U Sjevernoj Hrvatskoj. 1954;24:293-5.

[84] Richter S. Vrste Haemosporidia konja i goveda u NR Hrvatskoj njihova raširenost i vrijeme pojavljivanja. PhD thesis. Zagreb: University of Zagreb; 1954.

[85] Gotić, J. Clinical and serological Diagnostics and molecular typing of equine piroplasmosis aetiological agents in the Republic of Croatia. PhD thesis. Zagreb: University of Zagreb; 2015.

[86] Dežđek D, Vojta L, Ćurković S, Lipej Z, Mihaljević Ž, Cvetnić Ž, et al. Molecular detection of *Theileria**annae* and *Hepatozoon**canis* in foxes (*Vulpes**vulpes*) in Croatia. Vet Parasitol. 2010;172:333–6. 20646832 10.1016/j.vetpar.2010.05.022

[87] Beck A, Huber D, Polkinghorne A, Kurilj AG, Benko V, Mrljak V, et al. The prevalence and impact of Babesia canis and *Theileria* sp. in free-ranging grey wolf (Canis lupus) populations in Croatia. Parasit Vectors. 2017;10:1–9. 28376903 10.1186/s13071-017-2106-8 PMC5379697

[88] Pintur, K. Genetic Characterization of piroplasms infecting cervids in Croatia. PhD thesis, Zagreb: University of Zagreb; 2012.

[89] Laušević D, Ilić T, Nenadović K, Bacić D, Obrenović S. Seroprevalences of *Rickettsia**conorii*, *Ehrlichia**canis* and *Coxiella**burnetii* in Dogs from Montenegro. Acta Parasitol. 2020;65:271. 31848843 10.2478/s11686-019-00152-7

[90] Royal Vet Veterinary Clinic. Annual work reports. Podgorica: Royal Vet Veterinary Clinic; 2023

[91] Bajer A, Beck A, Beck R, Behnke JM, Dwuznik-Szarek D, Eichenberger RM, et al. Babesiosis in southeastern, central and northeastern Europe: an emerging and re-emerging tick-borne disease of humans and animals. Microorganisms. 2022;10:945. 35630388 10.3390/microorganisms10050945 PMC9146636

[92] Diagnostic Veterinary Laboratory. Annual work reports (2007–2023).

[93] Knuth P, Behn P, Schulze P. Experiments on equine piroplasmosis (Biliary Fever) in 1917. Z Vet besondere Berucksichtigung Hyg. 1918;6:241–64.

[94] Mekuli E. Contribution to the knowledge of the prevalence of parasitic diseases of domestic animals in Kosovo and Metohija. Vet Gl. 1958;6:356–8.

[95] Marković D. Parasitic diseases in Southern Serbia, the importance of their eradication for the production and human health. Jug Vet Gl. 1933;4:119–24.

[96] Pavlov P. The occurrence of theileriasis in Macedonia. Dtsch Tierarztl Wochenschr. 1942;50:43–4.

[97] Angelovski T. Some data about the most important invasive diseases in P.R. Macedonia Vet Gl. 1955;5:356–8.

[98] Angelovski T. Contribution to the knowledge of the piroplasmosis in Macedonia. Acta Vet. 1957;7:365.

[99] Angelovski T. About an epizooty of piroplasmosis in imported merino sheep in the area of Vardar-Chiflik near Negotino (at river Vardar). Vet Gl. 1958;1:21–2.

[100] Angelovski T, Petrović Z. Tomceva DSheep piroplasmosis in SR Macedonia. Vet Gl. 1963;10:861–7.

[101] Geru N. Sheep piroplasmosis in the municipality of Krushevo. Specialisation thesis. Belgrade: Veterinary Faculty; 1985 (in Serbian).

[102] Geru N, Cvetković LJ. Goat babesiosis in the region of Skopje. Macedonia Vet Rev. 1987;16:47–52.

[103] Geru N. Parasites in goats in Republic of Macedonia with special retrospection on babesiosis. PhD thesis. Skopje: Veterinary Faculty; 1996 (in Macedonian).

[104] Pavlović I, Milutinović M, Petković D, Terzin D, Terzin V. Epizootiological research of canine babesiosis in the Belgrade district. J Protozool Res. 2003;12:10–5.

[105] Mihaljica D, Radulović Ž, Tomanović S, Ćakić S, Penezić A, Milutinović M. Molecular detection of *Babesia* spp. in ticks in northern Serbia. Arch Biol Sci. 2012;64:1591–8.

[106] Tomanović S, Chochlakis D, Radulović Ž, Milutinović M, Ćakić S, Mihaljica D, et al. Analysis of pathogen co-occurrence in host-seeking adult hard ticks from Serbia. Exp App Acarol. 2013;59:367–76. 10.1007/s10493-012-9597-y 22773070

[107] Potkonjak A, Gutiérrez R, Savić S, Vračar V, Nachum-Biala Y, Jurišić A, et al. Molecular detection of emerging tick-borne pathogens in Vojvodina Serbia. Ticks Tick Borne Dis. 2016;7:199–203. 26565929 10.1016/j.ttbdis.2015.10.007

[108] Sukara R, Chochlakis D, Ćirović D, Penezić A, Mihaljica D, Ćakić S, et al. Golden jackals (*Canis**aureus*) as hosts for ticks and tick-borne pathogens in Serbia. Ticks Tick Borne Dis. 2018;9:1090–7. 29678402 10.1016/j.ttbdis.2018.04.003

[109] Davitkov D, Terzić S, Davitkov D, Radaković M, Gajić B, Krstić V, et al. Molecular detection of *Babesia* spp. in ticks sampled from asymptomatic dogs in the area of some Belgrade municipalities. Vet Glas. 2016;70:175–84.

[110] Banović P, Díaz-Sánchez AA, Galon C, Foucault-Simonin A, Simin V, Mijatović D, et al. A one health approach to study the circulation of tick-borne pathogens: a preliminary study. One Health. 2021;13:1–7. 10.1016/j.onehlt.2021.100270 PMC8188046 34141849

[111] Babić I. An overview of the development of Yugoslav medical (human and veterinary) parasitology until 1960 and its further basic tasks. Rev Yugosl Acad Sci Arts. 1965;10:160–74.

[112] Krstić V, Trailović D, Andrić N, Čalić M, Jovanović M. Contribution to epizootiology of dog babesiosis in Belgrade area. In: Proceedings of the 7th Conference of Veterinarians of Serbia, 13–16 September 1994, Zlatibor. p. 25–6.

[113] Bajer A, Beck A, Beck R, Behnke JM, Dwużnik-Szarek D, Eichenberger RM, et al. Babesiosis in southeastern, central and northeastern europe: an emerging and re-emerging tick-borne disease of humans and animals. Microorganisms. 2022;10:945. 35630388 10.3390/microorganisms10050945 PMC9146636

[114] Pavlović I, Antić V, Terzin V, Petković D, Terzin D, Ćurčin L, et al. Canine babesiosis in Belgrade area in period 2015–2017. In: Proceedings of the Workshop on Arthropod-Borne Diseases Transmitted by Ticks, Mites, Fleas, and Lice, 15–16 November 2018, Greifswald.

[115] Savić S, Vidić B, Grgić Z, Potkonjak A, Spasojevic LJ. Emerging Vector-Borne Diseases Incidence through Vectors. Front Public Health. 2014;2:267. 25520951 10.3389/fpubh.2014.00267 PMC4251170

[116] Davitkov D, Vucicevic M, Stevanovic J, Krstic V, Tomanovic S, Glavinic U, et al. Clinical babesiosis and molecular identification of *Babesia**canis* and *Babesia**gibsoni* infections in dogs from Serbia. Acta Vet Hun. 2015;63:199–208. 10.1556/AVet.2015.017 26051258

[117] Kovačević Filipović MM, Beletić AD, Ilić Božović AV, Milanović Z, Tyrrell P, Buch J, et al. Molecular and serological prevalence of* Anaplasma phagocytophilum*, *A**platys*, *Ehrlichia**canis*, *E*. *chaffeenses*, *E**ewingii*, *Borrelia**burgdorferi*, *Babesia**canis*, *B**gibsoni* and *B**vogeli* among clinically healthy outdoor dogs in Serbia. Vet Parasitol Reg Stud Rep. 2018;14:117–22. 10.1016/j.vprsr.2018.10.001 31014716

[118] Gabrielli S, Otasevic S, Ignjatovic A, Savic S, Fraulo M, Arsic-Arsenijevic V, et al. Canine babesioses in noninvestigated areas of Serbia. Vector Borne Zoonotic Dis. 2015;15:535–8. 26348245 10.1089/vbz.2015.1797

[119] Potkonjak A, Vračar V, Novakov N, Stevančević O, Stojanac N, Savić S, et al. Seroepidemiological research of babesiosis in dogs in the area of Novi Sad, Autonomous Province of Vojvodina, Republic of Serbia. J Vet Med Biotechnol Biosafe. 2015;12:22–4.

[120] Spasojević-Kosić LJ, Savić S, Potkonjak A, Vračar V. Retrospective analysis of clinical and laboratory findings in hunting dogs with serologic reactions to tick-borne pathogens (*Anaplasma**phagocytophilum*, *Borrelia**burgdorferi*, *Babesia**canis*, *Ehrlichia**canis*, *Ricketsia**conorii*). Vet Glas. 2015;69:219–32.

[121] Janjić F, Sarvan D, Tomanović S, Ćuk J, Krstić V, Radonjić V, et al. A short-term and long-term relationship between occurrence of acute canine babesiosis and meteorological parameters in Belgrade, Serbia. Ticks Tick Borne Dis. 2019;10:101273. 31445876 10.1016/j.ttbdis.2019.101273

[122] Potkonjak A, Savić S, Spasojević Kosić LJ, Tasić Otašević S, Tomanović S, Kovacevic FM. Consensus statement on epidemiological situation and expected frequency of canine vector-borne diseases in Serbia. Vet Glas. 2020;74:211–5.

[123] Ilić Božović A, Radaković M, Sparisou K, Phyllis T, Ramaswamy C, Mišić D, et al. First confirmed clinical case of *Anaplasma**platys* in a dog in Serbia. Acta Vet. 2021;71:107–12.

[124] Sukara R, Andrić N, Andrić JF, Mihaljica D, Veinović G, Ranković V, et al. Autochthonous infection with *Ehrlichia**canis* and *Hepatozoon**canis* in dogs from Serbia. Vet Med Sci. 2023;9:111–8. 36580396 10.1002/vms3.1061 PMC9857103

[125] Pavlović I, Zdravković N, Radanović O, Dobrosavljević I, Bojkovski J, Stokić-Nikolić S. Results of the research on blood parasites in cattle in Serbia. Int J Agric. 2022;10:445–50.

[126] Dschunkovsky E, Urodschevich V. Theileriasis in goats, sheep and cattle, with a description of* Theileria hirci* n.sp. from Serbia. Parasitology. 1924;16:107–10.

[127] Pavlović I, Dimitrijević A. II. Theileriosis in cow—a case report. In: International Agricultural, Biological and Life Science Conference, 7–9 July 2020, Edirne. p. 1219–21.

[128] Davitkov D, Davitkov D, Vucicevic M, Stanisic L, Radakovic M, Glavinic U, et al. A molecular and haematological study of *Theileria**equi* in Balkan donkeys. Acta Vet Hun. 2017;65:234–41. 10.1556/004.2017.023 28605963

[129] Vasić A, Nieder M, Zdravković N, Bojkovski J, Bugarski D, Pavlović I, et al. Tick infestation and occurrence of *Anaplasma**phagocytophilum* and piroplasms in cattle in the Republic of Serbia. Parasitol Res. 2018;117:1813–8. 29679202 10.1007/s00436-018-5867-x

[130] Juwaid S, Sukara R, Penezić A, Mihaljica D, Veinović G, Kavallieratos NG, et al. First evidence of tick-borne protozoan pathogens, Babesia sp. and* Hepatozoon canis*, in red foxes (*Vulpes vulpes*) in Serbia. Acta Vet Hung. 2019;67:70–80. 30922092 10.1556/004.2019.008

[131] Duscher G, Ćirović D, Heltai M, Szabó L, Lanszki J, Bošković I, et al. Hepatozoonosis in Golden Jackals (*Canis aureus*) from Southeastern and Central Europe: prevalence data from a rirst molecular screening. In: First International Jackal Symposium, 13–16 October 2014, Veliko Gradište. p. 70–1.

[132] Kuručki M, Tomanović S, Sukara R, Ćirović D. High prevalence and genetic variability of hepatozoon canis in grey wolf (*Canis lupus* L. 1758) population in Serbia. Animals. 2022;12:3335. 36496856 10.3390/ani12233335 PMC9740517

[133] Veinović G, Sukara R, Mihaljica D, Penezić A, Ćirović D, Tomanović S. The occurrence and diversity of tick-borne pathogens in small mammals from Serbia. Vector Borne Zoonotic Dis. 2024;24:285–92. 38346321 10.1089/vbz.2023.0088

[134] Geiger J, Morton BA, Jose E, Vasconcelos R, Tngrian M, Kachani M, et al. Molecular characterization of tandem repeat protein 36 gene of *Ehrlichia**canis* detected in naturally infected dogs from Peru. Am J Trop Med Hyg. 2018;146:1–13. 10.4269/ajtmh.17-0776 PMC6090345 29943707

[135] Bouza-Mora L, Dolz G, Solórzano-Morales A, Romero-zu JJ, Salazar-Sánchez L, Labruna MB, et al. Novel genotype of *Ehrlichia**canis* detected in samples of human blood bank donors in Costa Rica. Ticks Tick-Borne Dis. 2016;8:36–40. 27682202 10.1016/j.ttbdis.2016.09.012

